# *Aedes aegypti dyspepsia* encodes a novel member of the SLC16 family of transporters and is critical for reproductive fitness

**DOI:** 10.1371/journal.pntd.0009334

**Published:** 2021-04-07

**Authors:** Hitoshi Tsujimoto, Michelle A. E. Anderson, Heather Eggleston, Kevin M. Myles, Zach N. Adelman

**Affiliations:** 1 Department of Entomology, Texas A&M Agrilife Research, College Station, Texas, United States of America; 2 Department of Entomology, Virginia Tech, Blacksburg, Virginia, United States of America; University of Glasgow, UNITED KINGDOM

## Abstract

As a key vector for major arthropod-borne viruses (arboviruses) such as dengue, Zika and chikungunya, control of *Aedes aegypti* represents a major challenge in public health. Bloodmeal acquisition is necessary for the reproduction of vector mosquitoes and pathogen transmission. Blood contains potentially toxic amounts of iron while it provides nutrients for mosquito offspring; disruption of iron homeostasis in the mosquito may therefore lead to novel control strategies. We previously described a potential iron exporter in *Ae*. *aegypti* after a targeted functional screen of ZIP (zinc-regulated transporter/Iron-regulated transporter-like) and ZnT (zinc transporter) family genes. In this study, we performed an RNAseq-based screen in an *Ae*. *aegypti* cell line cultured under iron-deficient and iron-excess conditions. A subset of differentially expressed genes were analyzed via a cytosolic iron-sensitive dual-luciferase reporter assay with several gene candidates potentially involved in iron transport. *In vivo* gene silencing resulted in significant reduction of fecundity (egg number) and fertility (hatch rate) for one gene, termed *dyspepsia*. Silencing of *dyspepsia* reduced the induction of ferritin expression in the midgut and also resulted in delayed/impaired excretion and digestion. Further characterization of this gene, including a more direct confirmation of its substrate (iron or otherwise), could inform vector control strategies as well as to contribute to the field of metal biology.

## Author summary

*Aedes aegypti* is the major vector of dengue, Zika, chikungunya and yellow fever viruses. One of the most effective ways to reduce transmission of these viruses is to control *Ae*. *aegypti* populations. However, these mosquitoes continue to develop resistance to conventional insecticides. Therefore, alternative methods to control mosquitoes are urgently needed. Most mosquitoes require a bloodmeal greater than their body weight in order to produce eggs. The nutrient-rich blood also contains a large amount of iron, which in such large quantities could be toxic. This implies that the regulation of iron homeostasis is critical to mosquito fitness, and suggests that the mechanisms for how mosquitoes achieve this balancing act with regards to iron may lead to novel mosquito control approaches. Here, we performed a screen to identify transporters potentially involved in mosquito iron homeostasis. After identifying differentially expressed genes via RNAseq and validating a subset in cell culture assays, gene silencing experiments in whole mosquitoes revealed a gene whose reduction resulted in a change in ferritin gene expression and a drastic reduction of egg numbers and hatch rate. Further investigation of this gene may lead to novel mosquito control approaches.

## Introduction

As a key vector of arboviruses such as dengue [1], Zika [2], yellow fever [3] and chikungunya [4] viruses, the global expansion of *Aedes aegypti* has played a leading role in the corresponding expansion of these arboviruses, and has fueled epidemics of international concern [5]. Thus, controlling *Ae*. *aegypti* represents a major pathway to reducing the transmission of medically important arboviruses. As chemical (insecticide) control is being hampered by the emergence of resistance [6], alternative ways to control vector populations are urgently needed.

Arboviruses are transmitted when a female mosquito takes our blood, which is required to complete egg development. While vertebrate blood provides mosquitoes with protein-rich nutrients to mature eggs, it also contains a large amount of iron that can be detrimental to the mosquitoes [7]. During the catalysis of hemoglobin (the major blood protein), heme and free iron are released and are transported, sequestered or degraded to avoid damaging cellular components via their oxidative properties [7]. Despite iron being toxic at high concentrations, it is also an essential nutrient as many enzymes required in key biological functions contain iron as a co-factor, such as iron-sulfur (Fe-S) clusters in the electron transport chain in oxidative metabolism [8,9], implying the need for tight regulation of iron in mosquitoes. Moreover, oxidative stress caused by iron could control viral infection in mosquitoes, as a recent study showed that host plasma iron level affected dengue virus infection in *Ae*. *aegypti* [10]. Xanthurenic acid produced by tryptophan metabolism is known to protect the midgut epithelium by binding to iron and heme to inhibit lipid peroxidation [11]. Hence, tipping the mosquito’s iron homeostasis to create a slight imbalance may drastically affect the mosquito’s physiology, which may lead to novel mosquito and transmission control strategies.

The transport of iron in mosquitoes, even in insects in general is not well understood, with the exception of ferritins [7,12]. Ferritin is an iron-storage protein consisting of 24 hetero-multimeric subunits (12 heavy chains and 12 light chains) that makes a hollow sphere to cage up to 4500 Fe^3+^ [13]. Unlike vertebrate ferritin, which principally stores iron in the cytoplasm, insect ferritin is found in the secretory compartments and is considered to transport iron via the circulatory system, as antiserum specific to *Ae*. *aegypti* ferritin subunits [14] detected ferritin from adult mosquito hemolymph [15]. The route and molecular mechanism of acquiring iron prior to packaging into ferritin, however, remains to be elucidated. To our knowledge, the divalent metal ion transporter NRAMP (natural resistance associated macrophage protein) and ZIP13 are the only free-iron transporters described in insects. A variety of organisms from bacteria to human possess NRAMP homologs, which transport iron (Fe^2+^) among other divalent metals [16]. A mutant of the NRAMP homolog, *Malvolio* (*Mvl*), in *Drosophila melanogaster* showed defects in sugar tasting, which was rescued by supplementation of Mn^2+^ and Fe^2+^ [16], suggesting Mvl has iron-transporting function. Notably, AnaNRAMP from the American malaria mosquito, *Anopheles albimanus* is the only insect NRAMP biochemically verified for iron-transporting function [17]. In *D*. *melanogaster*, dZIP13 has been determined to be an iron exporter by transporting iron from the cytoplasm to the endoplasmic reticulum (ER) suggesting its key role in loading iron into ER for packaging in ferritin [18]. Interestingly, dZIP13 seems to operate in an unconventional direction (outward) to transport substrate (iron) [19].

Surprisingly, no gene closely related to any NRAMP homologs has been found in the mosquitoes in the subfamily Culicinae. In a search for genes that encode an NRAMP-like function we identified potential iron transporters from both the ZIP (zinc-regulated transporter/Iron-regulated transporter-like) and ZnT (zinc transporter) families in *Ae*. *aegypti*. In particular, silencing of AaeZIP13 showed increased iron accumulation in the midgut and reduced iron in the ovaries at 24 h post bloodmeal as expected from the results for dZIP13 in *D*. *melanogaster*. However, the effect did not persist, as once egg maturation was complete we observed no difference in fecundity and fertility [20].

Thus, the study we describe in this report was designed and conducted to find additional iron transporters which may play important roles in iron homeostasis in *Ae*. *aegypti*. We performed an RNAseq screen for iron transporters using an *Ae*. *aegypti* cell line (Aag2) exposed to excess and depleted iron conditions. Genes further selected by an iron-sensitive luciferase reporter assay were tested by *in vivo* gene silencing. One gene, when silenced, showed a drastic reduction in fecundity and fertility as well as ferritin expression in the midgut. Sequence analysis of this gene indicated orthologs in mosquitoes are strikingly conserved, suggesting similar functions within vector mosquitoes.

## Materials and methods

### Mosquitoes and mosquito cell culture

*Aedes aegypti* Aag2 and A20 cells were maintained in Leibovitz’s L-15 (L15) medium (Thermo Fisher) supplemented with 10% FBS (Atlanta Biological), 2% tryptose phosphate broth (Thermo Fisher) and 1% penicillin/streptomycin (hereafter “complete” L15 = cL15) at 28°C in closed flasks. *Ae*. *aegypti* (“Liverpool” strain) mosquitoes were maintained in an insectary in environmental chambers kept at 27°C, 80% RH and 14:10 (L:D) cycles. Immature stages were reared with ground TetraMin (Tetra) in 49.5 × 29.2 × 8.9 cm translucent trays with 2 L of water at 500 larvae/tray density (2.89 cm^2^ or 4 mL per larva), adults were maintained with 10% sucrose and females were fed on defibrinated sheep blood (Colorado Serum Company) for egg production. To examine organ-specific transcript expression, midguts (Mg), Malpighian tubules (MT), ovaries (Ov) and carcasses (whole body without Mg, MT and Ov: C) were dissected from female mosquitoes in 1× PBS and transferred immediately into 1.5-mL tubes containing TRIzol reagent (Thermo Fisher). Samples were taken from sugar-fed (5 days after eclosion), 6 h post bloodmeal (6 hPBM) and 24 hPBM from triplicated batches (20–30 per batch).

### RNAseq analysis of cultured cells

Aag2 cells were seeded in triplicate T-25 flasks and then the following day the medium was replaced with either cL15 without any FBS, cL15 without FBS supplemented with 50 μM deferoxamine (DFO), or cL15 without FBS supplemented with 100 μM ferric ammonium citrate (FAC). After 48 h cells were harvested and RNA was extracted using TRIzol (Life Technologies), according to the manufacturer’s instructions. Libraries were prepared using the NEBNext Ultra RNA Library Prep Kit for Illumina using the NEBNext Poly(A) mRNA Magnetic Isolation Module (New England Biolabs, NEB) and multiplexed into 3 lanes of an Illumina HiSeq 2500. Reads were aligned to the *Ae*. *aegypti* reference genome (AaegL3 and AaegL5, obtained from www.vectorbase.org [21]) using the default parameters of HISAT2 v2.1.0 [22] through the Texas A&M’s High Performance Research Computing Ada server. Low quality mapping scores were removed from sorted mapped reads using SAMtools suite v1.7 [23]. BEDtools suite v2.19.1 [24] was utilized to count the number of reads per mRNA transcript. Differential expression analysis was performed using the exact test [25] as implemented in edgeR [26]. Transmembrane domains of the differentially expressed genes were predicted by the TOPCONS webserver [27]. MA plots were generated using the “ggplot2” package [28] on R [29].

### Quantitative real-time PCR (qRT-PCR)

Total RNA was isolated using TRIzol (Thermo Fisher) and treated by DNase (TURBO DNA*free* kit, Thermo Fisher). cDNA was synthesized using SuperScript IV (Thermo Fisher) and oligo d(T)_20_-VN primers with 1 μg of RNA in 20 μL reaction by incubation at 65°C for 5 min, chilled on ice for > 1 min, 50°C for 50 min and 80°C for 10 min. cDNA was diluted to 1/50 for qRT-PCR templates. Primer3 [30] was used to design primer pairs. Prior to using Primer3, the target sequence was subjected to Mfold [31] to predict secondary structures at the expected annealing temperature (60°C) to exclude such regions from primer annealing sites. The primers were empirically verified for optimal annealing temperature range and amplification efficiency (E) using serial dilution of cDNA templates. All primer pairs were determined to have E between 90–111 (in %). All primers and E are listed in S1 File (qRT-PCR tab). qRT-PCR reactions were run on a Bio Rad CFX96 using SsoAdvanced Universal SYBR Green Supermix (Bio Rad). Amplification parameters were 95°C for 30 s and 45 cycles of 95°C for 15 s and 57°C for 30 s followed by melt analysis between 65–95°C. Ribosomal protein S7 gene was used to normalize expression. All reactions were performed in triplicate.

### RNAi gene silencing in cells and mosquitoes

dsRNA and associated primers were designed using the E-RNAi web server [32]. Since the reference genome used in E-RNAi for *Ae*. *aegypti* was out of date (AaegL1.2), we performed a blastn search against the AaegL5.1 geneset (vectorbase.org, [21]) by local blast+ (ver 2.6.0) with “-word_size 19” option using the entire length of dsRNA as query to search off-target transcripts. All the primers are listed in S1 File. We only accepted dsRNA that did not have any hits against any gene except for itself. Templates were amplified by PCR using Phusion DNA polymerase (NEB) with primers that have T7 promoter sequence at the 5’ end. PCR products were purified by NucleoSpin Gel and PCR cleanup (Machery-Nagel). dsRNA synthesis was performed using MEGAScript T7 kit (Thermo Fisher) using 1 μg of template. The reaction was treated by DNase and purified by MEGAClear kit (Thermo Fisher). Resultant dsRNA was quantified at 260 nm absorbance on a SpectraMax i3x (Molecular Devices). dsRNA for cell culture assays were diluted to 640 ng/μL and 5 μL (3.2 μg) aliquots were made and stored at −80°C. For adult injection, aliquots of volumes equivalent to 25 injections (1 μg/mosquito) were made from undiluted dsRNA suspension and stored at −80°C. As a control, dsRNA against EGFP (dsEGFP) was also made. For adult gene silencing, we used females at 1 day after eclosion, with 1 μg of dsRNA injected into each female mosquito through the thorax using Nanoject II (Drummond) with a borosilicate capillary needle pulled by a Micropipette puller (Sutter P-2000). Injected females were kept in the presence of males to ensure mating for 3 days and were bloodfed using artificial feeders and citrated sheep blood (Hemostat); only engorged females were kept.

### Dual-luciferase plasmid construction

A gene cassette containing the *Ae*. *aegypti* polyubiquitin promoter-*Renilla* luciferase (PUb-RL) was excised from pSLfa-PUb-RL [33] using MluI and EcoRI (NEB) and treated with mung bean nuclease (NEB) to make the ends blunt. A separate plasmid containing the *Ae*. *aegypti* ferritin light chain (AAEL007383) promoter-firefly luciferase (FerLCH-FFL) [20] gene was linearized 3’ of the FFL ORF using PshAI and treated with shrimp alkaline phosphatase (NEB). The PUb-RL fragment was ligated into the linearized pGL3-LCH-FFL, and only tail-to-tail orientation constructs were sequenced to confirm the direction and integrity of the insert. A sequence-confirmed clone was purified using the endotoxin-free Midiprep (NucleoBond Xtra Midi EF, Machery-Nagel) for transfection.

### Cell culture gene silencing and dual luciferase assay

Cells at confluency were detached by flushing with fresh cL15 using a 10-mL pipette about 15 times and scraping (Aag2 cells) or scraping alone (A20 cells), passed through a 10-mL serological pipette tip for 20 times to dissociate clumps and counted using the trypan blue viability method. For each experiment, 3 × 10^6^ cells (Aag2) or 1 × 10^6^ cells (A20) were seeded in each well of 6-well plates filled with 2 mL of fresh cL15 in each well and incubated at 28°C in a sealed container. After 3 days of culture when cells reached confluency, the medium was replaced with 2 mL of fresh cL15 without antibiotics (cL15noAb). For transfection, 200 μL of opti-MEM I reduced serum medium (opti-MEM, Thermo Fisher) was mixed with 5 μL of dsRNA aliquot (3.2 μg), and another 200 μL of opti-MEM was mixed with 8 μL of Lipofectamine 2000 (L2000, Thermo Fisher). The dsRNA mix and L2000 mix were combined and incubated at room temperature for 5 min, and this mixture was added to each well of the 6-well plate and incubated at 28°C in a closed container. On the next day, half of the medium (1 mL) was replaced with fresh cL15noAb to reduce toxicity of the transfection reagent. Medium was replaced likewise on the second day as well. On the third day, all medium was removed from each well and replaced with 1 mL of fresh cL15noAb. Cells were detached by repeated pipetting using a 1000 μL pipette, counted using trypan blue and adjusted to 5 × 10^6^ cells/mL. One hundred microliters (100 μL) of the cell suspension was seeded in each well of an entire column (8 wells) of a 96-well tissue culture plate.

For dual-luciferase plasmid transfection, 100 ng of plasmid was mixed with 25 μL of opti-MEM, and 0.5 μL of L2000 was mixed with 25 μL of opti-MEM. The two mixes were combined, incubated at room temperature for 5 min and 50 μL was transferred to each well of the 96-well plate. The plate was incubated at 28°C in a sealed container. At 24 h after transfection, 50 μL of 400 μM FAC in cL15noAb was added in each well (final concentration: 100 μM FAC each well) and incubated at 28°C in a sealed container. On the next day, the medium was aspirated and cells were washed with 100 μL of 1× phosphate-buffered saline (PBS). PBS was removed and 45 μL of 1× Passive lysis buffer (Dual-Luciferase Reporter assay kit, Promega) was added to each well and incubated at room temperature for 15 min on a plate shaker (advanced microplate vortex mixer, VWR) at 600 rpm. Twenty microliters (20 μL) of the lysates were transferred to each well of a flat-bottom white 96-well plate (Greiner bio-one, ref 655083), and dual-luciferase assay was performed on a SpectraMax i3x with injector module (Molecular Devices) with 80 μL injection volume for both Luciferase Assay Reagent II and 1× Stop & Glo reagent with 10 s read time and 2 s interval. The entire assay was replicated 3–5 times per target gene.

Since the normalized luminescence values (firefly/*Renilla*) differed between assay dates, the values were further normalized to the control (dsEGFP) and log_2_ transformed. All assay data were combined and analyzed by a mixed effect model using R (lme4 package) by the formula: y ~ gene + (1|day), where “y” is the normalized values, “gene” is the target of dsRNA and “day” is the assay day, which assumes the effect of dsRNA is under the random effect of assay days.

Graphs were plotted using GraphPad Prism 7 (GraphPad Software).

### Fecundity and fertility assay

Fecundity and fertility were examined using the EAgaL plate assay used in the previous study [20] and described in detail in [34]. Briefly, dsRNA-injected and starved-overnight females were bloodfed at 3 days post injection on citrated sheep blood (Hemostat) and only engorged females were kept in a container with 30% sucrose (to prevent accidental egg laying on the sucrose water). After 72 h, the mosquitoes were transferred to 24-well plates with 2% agarose layer on the bottom of each well. Females were allowed to lay eggs for 48 h and each well was photographed for egg counting using a TG-4 compact digital camera (Olympus). After photographing, water was added to each well and incubated for 7 days in the environmental chamber at 27°C. Hatched larvae in each well were photographed for counting upon anesthesia on ice. Counting of eggs and larvae was performed using ImageJ (Fiji) software [35]. The experiment was independently repeated three times. Obtained data were analyzed using Graphpad Prism 7.

### Membrane topology prediction and sequence alignments

Membrane topology of AAEL000471 and homologues was predicted by TOPCONS web server (http://topcons.cbr.su.se) [27], with graphics made by Protter [36] using TOPCONS topology. Human SLC16 homologs were retrieved from GenBank [37] (S1 File). The amino acid sequence of AAEL000471 was used as a query in a blastp search of the NCBI nr database and/or Vectorbase [21] to identify homologs in the fruit fly *D*. *melanogaster*, the African malaria mosquito *An*. *gambiae*, the zebrafish *Danio rerio* and in the yeast *Saccharomyces cerevisiae*, as well as paralogs in *Ae*. *aegypti*. Overall alignment of 86 protein sequences (S1 File) was performed in MEGA7 using Muscle [38]. The resulting alignment was used to generate a phylogenetic tree using the Neighbor-joining method. Alignments of AAEL000471 and human SLC16 genes or its closest paralogs in other dipterans were performed with Clustal Omega at EMBL-EBI [39], and manually marked TMDs and the cytoplasmic loop on Microsoft Word.

## Results

### Candidate selection utilizing cell culture RNAseq and dual-luciferase reporter assay

#### RNAseq analysis

**Fig 1:**
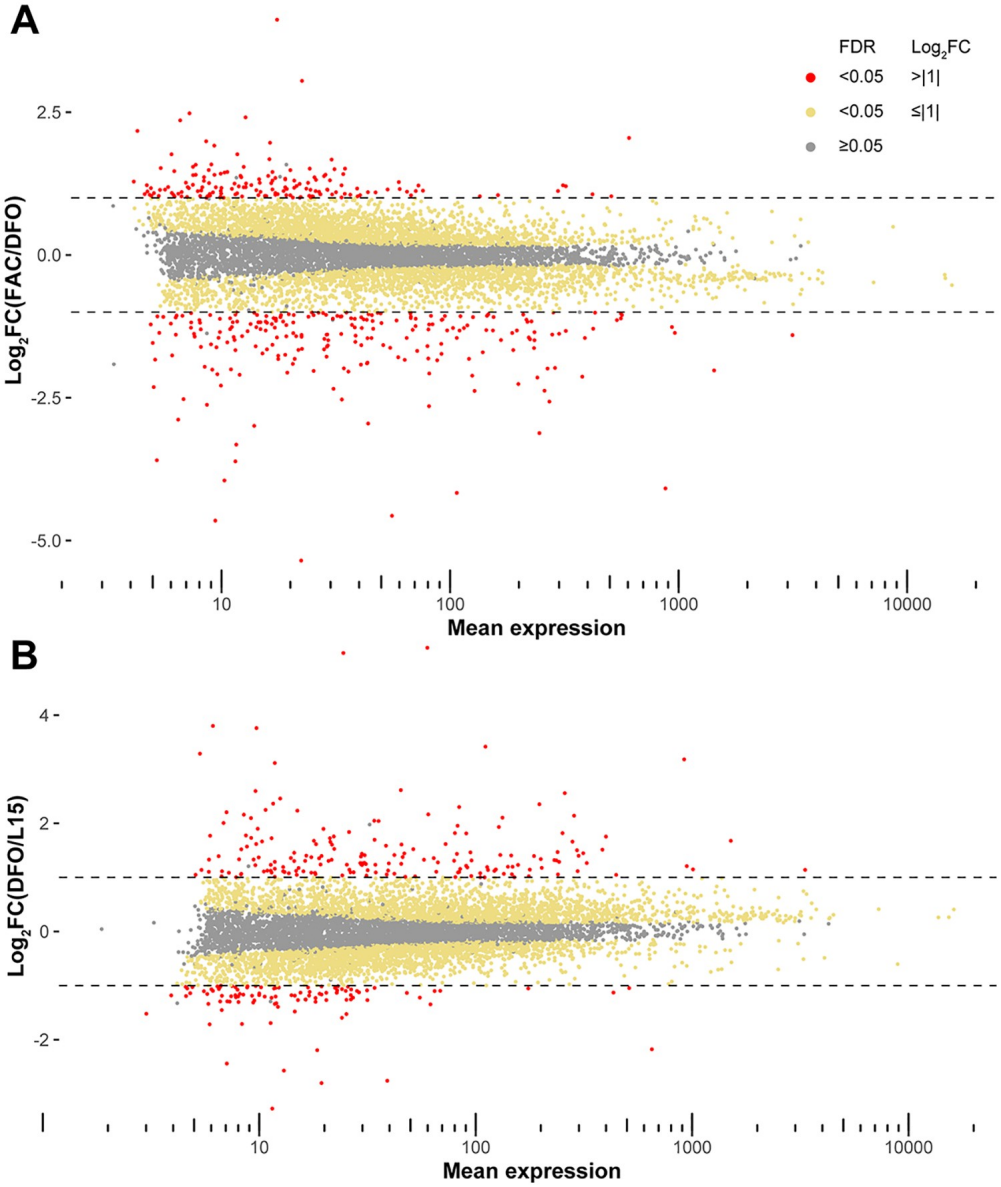
MA plots of expressed genes in Aag2 cells. (A) MA plot showing differentially expressed genes in Aag2 cells under FAC (100 μM) vs DFO (50 μM). (B) MA plot for the genes in Aag2 cells under DFO (50 μM) vs L15. Log_2_FC: log_2_-transformed fold change values; FDR: false discovery rate; grey dots: FDR ≥ 0.05; yellow dots: FDR < 0.05 and Log_2_FC < |1|; red dots: FDR < 0.05 and Log_2_FC ≥ |1|.

To find candidate iron transporters in *Ae*. *aegypti*, Aag2 cells cultured in the presence of excess iron (FAC 100 μM), iron-depleted (DFO 50 μM) and basal condition (L15 without FBS) were subjected to Illumina sequencing for differential gene expression analysis. Obtained reads were deposited to GEO (Gene Expression Omnibus) with the accession number: GSE160498. We initially mapped the reads onto the AaegL3 assembly, and after release of the AaegL5 version, which significantly improved the assembly into chromosomes [40], we re-mapped the reads onto the new assembly. We found that 4471 genes and 4461 genes were significantly different in transcript abundance (false discovery rate, FDR ≤ 0.05) between FAC vs DFO and DFO vs L15, respectively, when mapped to the AaegL5 assembly (Fig 1A and 1B). We further filtered the genes with the following criteria to obtain a candidate gene list: A) predicted to have ≥ 3 transmembrane domains, B) Log_2_ fold change equal or larger than 1 and equal or smaller than −1 (Log_2_FC ≥ |1|) for both DFO/L15 and FAC/DFO comparisons, C) annotated to be “unknown” or “transporter”. Because we performed this process for both AaegL3- and AaegL5-mapped differentially-expressed gene lists, we compared the two candidate gene lists. These lists contained 14 and 17 genes for AaegL3 and AaegL5 assemblies, respectively, of which 7 genes were present in both lists, 3 genes from AaegL3 were re-annotated to be non-membrane proteins and 4 genes were only found in the list of AaegL3, because one or both log_2_FC values did not pass the criterion B) when mapped onto AaegL5 genome (Table 1, blue fonts). We included these genes for downstream analysis. Among these 21 genes, 2 genes are almost identical in cDNA sequences (AAEL014972 and AAEL020524), and we targeted both with one dsRNA. Based on the fold change of expression, we assigned 4 genes as candidate exporters as the depletion of iron reduced and excess iron increased their expression, while the other 17 genes were considered as potential importers as their expression pattern was the opposite. Among some of the candidate genes, we observed another *Ae*. *aegypti* cell line, A20, exhibited higher expression than Aag2 [41], and we used A20 cells for these genes for the downstream dual luciferase assay (Table 1). The original differential expression tables for AaegL3 and AaegL5 mapped results and normalized counts for Aag2 and A20 cells are presented in S2 and S3 Files, respectively.

**Table 1:** Candidate iron transporter genes selected from RNAseq data. GeneID: VectorBase gene ID. Log_2_FC: log_2_ fold change between indicated comparison groups. in/ex: predicted iron transporting function; in: importer, ex: exporter. Cell line: cell line used for dual-luciferase reporter assay; AAEL013277 was used for both cell lines as the expression was high in both cell lines. Normalized count: normalized CPM (counts per million) for the two cell lines. There was only one dsRNA available for AAEL014972 and AAEL020524 (red fonts) due to high identity between these genes. AAEL008624 and AAEL012698 (grey-shaded) could not be analyzed downstream due to failure of synthesizing dsRNA. Genes in bold fonts are present in both the candidate tables from AaegL3 and AaegL5 assemblies. TMD: number of predicted transmembrane domains. Blue fonts in Log_2_FC indicates values lower than threshold in AaegL5 mapping, but higher in AaegL3 mapping. Superfamily/Domain: Predicted superfamily/domain by blastp. SLC: solute carrier. TMEM: transmembrane protein. MFS: major facilitator superfamily. ABC: ATP binding cassette. CLC: chloride channel. DUF: domain of unknown function.

		Log_2_FC				Normalized count (cpm)			
	GeneID	DFO/noFBS	FAC/DFO	in/ex	Cell line	Aag2	A20	TMD	Superfamily/Domain
1	AAEL019992	-1.69	1.76	ex	Aag2	17.24	NA	10	SLC35-like
2	AAEL000230	-1.16	1.19	ex	A20	7.93	10.69	3	TMEM126
3	**AAEL012109**	-1.13	1.26	ex	Aag2	21.26	NA	9	SLC45-like
4	AAEL020619	-1.10	1.11	ex	Aag2	88.11	NA	12	MFS transporter
5	AAEL000471	$\begin{array}{c} 0.88 \end{array}$	$\begin{array}{c} -0.90 \end{array}$	in	Aag2	65.20	32.89	12	SLC16-like
6	**AAEL008406**	1.05	-1.12	in	Aag2	25.27	NA	12	SLC7-like
7	AAEL008624	1.06	-1.43	in		5.34	2.06	6	ABC transporter-like
8	AAEL012698	$\begin{array}{c} 0.96 \end{array}$	-1.07	in		65.86	6.65	12	ABC transporter-like
9	**AAEL018228**	1.09	-1.30	in	Aag2	7.26	NA	11	SLC16-like
10	**$\begin{array}{c} AAEL014972 \end{array}$**	1.14	-1.32	in	Aag2	71.38	18.78	12	SLC2-like
11	$\begin{array}{c} \text{AAEL}020524 \end{array}$	1.14	-1.49	in	Aag2	111.26	7.31	12	SLC2-like
12	AAEL011520	1.23	-1.39	in	Aag2	17.87	NA	12	SLC45-like
13	**AAEL007936**	1.23	-1.32	in	Aag2	93.72	46.31	7	TMEM156
14	AAEL019918	1.07	$\begin{array}{c} -0.81 \end{array}$	in	Aag2	87.10	NA	6	voltage-gated K channel
15	**AAEL012522**	1.32	-1.48	in	Aag2	132.37	NA	10	SLC17-like
16	AAEL013277	$\begin{array}{c} 0.24 \end{array}$	$\begin{array}{c} -0.42 \end{array}$	in	A20/Aag2	3942.60	2108.10	20	Na channel
17	AAEL026712	1.41	-1.78	in	Aag2	16.78	4.09	3	NA
18	AAEL005950	1.68	-1.76	in	A20	8.55	228.15	12	CLC1-like
19	AAEL007758	1.78	-1.96	in	A20	3.93	50.14	5	DUF4149-like
20	**AAEL003135**	2.05	-1.98	in	A20	13.24	76.25	12	MFS transporter
21	AAEL006180	2.25	-2.00	in	A20	3.72	60.48	12	SLC12-like

#### Gene silencing and luciferase reporter assay to verify iron transport by the candidate genes

**Fig 2:**
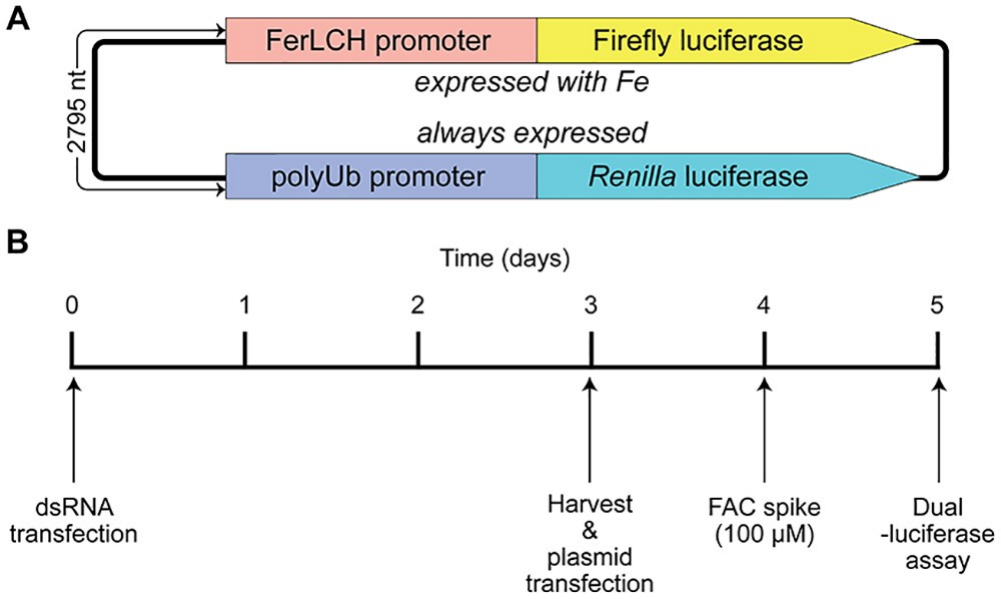
Graphical representation of dual-luciferase reporter plasmid and assay procedure. (A) Two luciferase reporter genes in a single plasmid in tail-to-tail orientation. FerLCH: *Ae*. *aegypti* ferritin light chain (AAEL007383). polyUb: *Ae*. *aegypti* poly-ubiquitin (AAEL003888). (B) Timeline for gene silencing and dual-luciferase assay procedure. FAC: ferric ammonium citrate.

We previously used a two-plasmid system based on the activation of the *Ae*. *aegypti* ferritin light chain promoter (FerLCH) to detect cytosolic iron levels in mosquito cells [20]. In this study, we combined these two luciferase cassettes (FerLCH-Firefly luciferase + PUb-Ren luciferase) into one plasmid (Fig 2A). To determine if any of the differentially expressed genes are involved in iron transport, we silenced each candidate gene in *Ae*. *aegypti* cells with dsRNA and assessed cytosolic iron levels using this dual luciferase reporter (Fig 2B).

**Fig 3:**
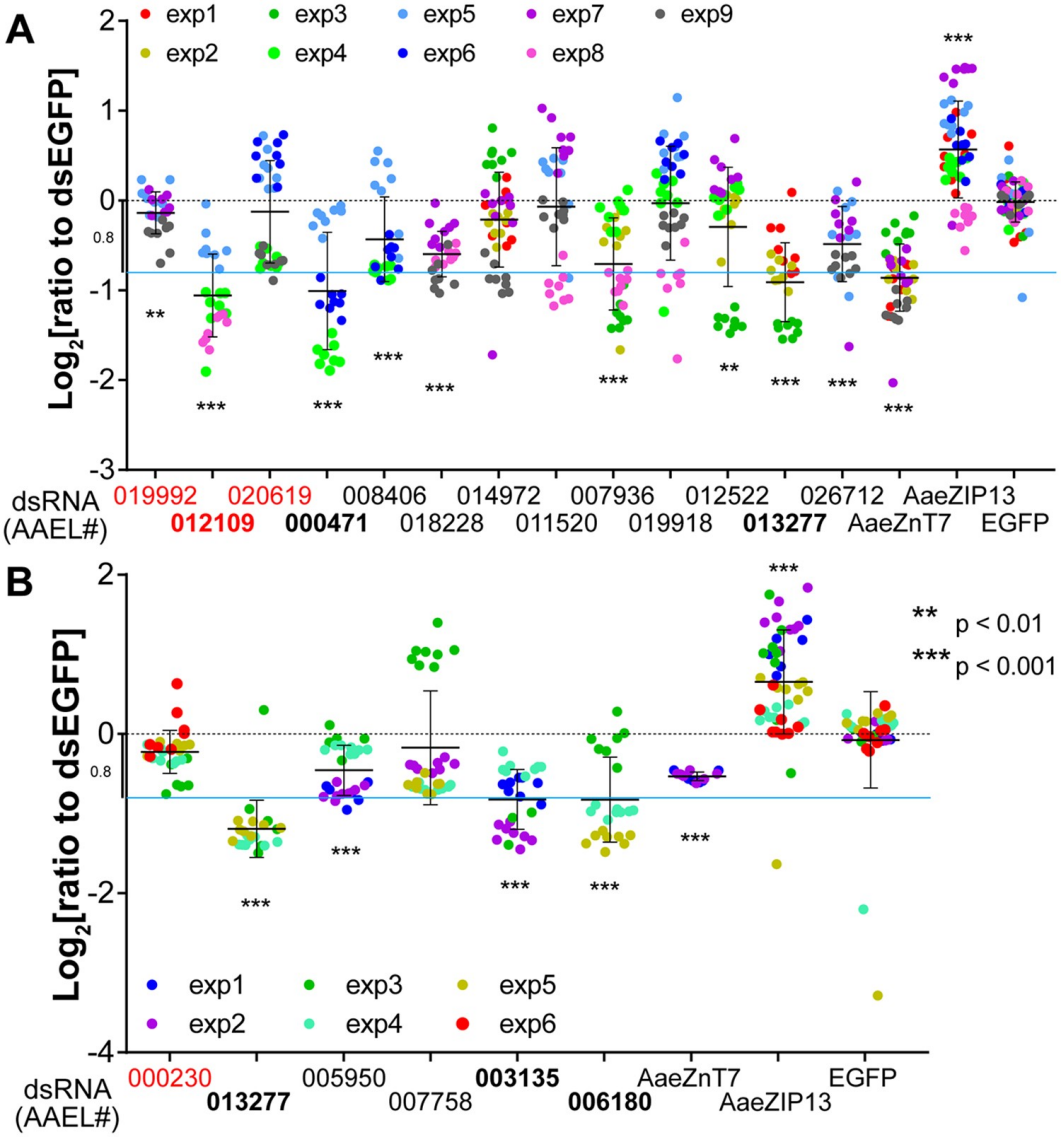
RNAi knockdown of candidate iron transporters changes iron-responsive reporter expression in mosquito cells. (A) Dual-luciferase assay in Aag2 cells and (B) in A20 cells. Normalized firefly luciferase luminescence values (to EGFP control) are plotted on a Log_2_ scale with mean ± SD. Different dot colors indicate individual experiments. Neutral level is indicated by a black dotted line; sky-blue line indicates manually set cutoff (−0.8log_2_) for downstream analysis. The targets of dsRNA (either VectorBase gene ID [AAEL number] or gene name) are on the x axis. The predicted exporters are indicated by red fonts and the five genes selected for downstream analyses are in bold fonts. Statistical significance by mixed effect model (see methods) between EGFP control and each sample are shown in the figure.

Since a single 96-well plate could not cover all the genes including controls under our protocol, we performed this assay with a reasonable number of target genes at a time. The assay per target gene was independently replicated 3–5 times (Fig 3A and 3B shown in dot colors). Inclusion of the previously described AaeZIP13 (potential exporter) and AaeZnT7 (potential importer) genes showed results consistent to our previous findings (Fig 3A and 3B) [20]. Amplification of dsRNA templates for AAEL008624 and AAEL012698 failed and could not be tested. When each candidate gene was silenced, we repeatedly observed a significant difference in reporter expression for several genes with respect to cells treated with dsRNA against EGFP (negative control). 10 of 15 tested importer candidates showed a significant decrease in the iron-responsive luciferase expression following dsRNA treatment. To our surprise, silencing of two candidate exporters also resulted in decreased reporter expression, one being a drastic reduction. From this, the five genes showing the strongest decrease in ferritin promoter-driven luciferase activation were selected for follow-up analysis *in vivo*.

#### Silencing of candidate gene AAEL000471 alters midgut ferritin levels after a bloodmeal

**Fig 4:**
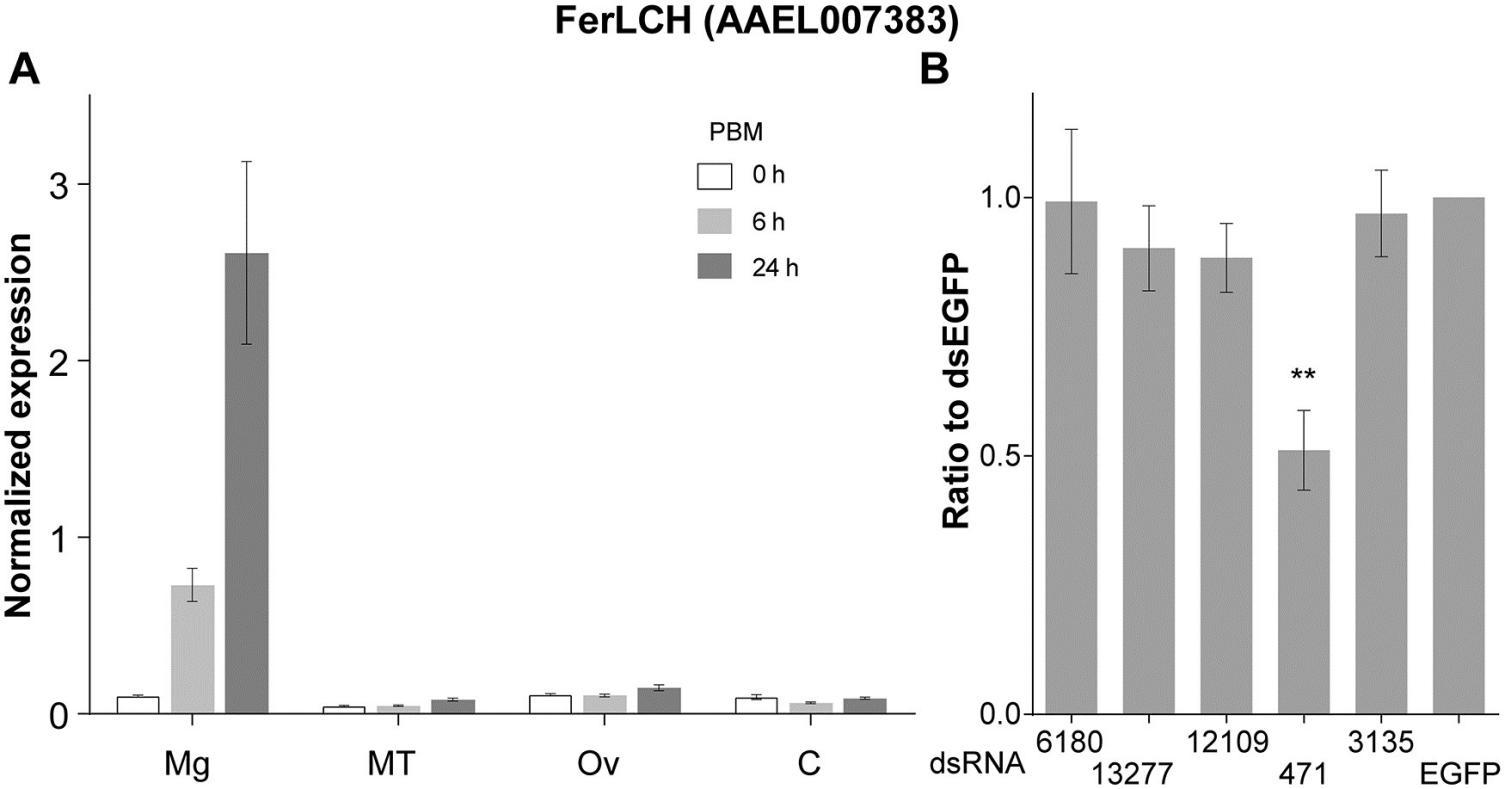
Ferritin light chain mRNA expression. (A) qRT-PCR quantification of transcript expression pre- and post-bloodmeal in the midgut (Mg), Malpighian tubules (MT), Ovaries (Ov) and remaining body parts (C) (mean±SD normalized to rpS7), (B) ferritin LCH expression in Mg 24 hPBM in dsRNA-injected mosquitoes (n = 8–12) (mean±SD of ratio to dsEGFP of rpS7 normalized expression). **: p < 0.01 by Kruskal-Wallis test followed by multiple comparison.

Similar to previous reports [42,43], we observed that transcription of FerLCH is strongly activated in the midgut following a bloodmeal (Fig 4A), presumably to control cytoplasmic iron levels and shuttle bloodmeal iron to the developing ovaries. If any of the candidate genes identified were involved in iron import during blood digestion, silencing of these genes should result in decreased cytoplasmic iron levels and hence a decrease in induced ferritin transcripts. With previous data showing these genes, except for AAEL012109 were expressed in the midgut [43], we performed RNAi gene silencing of each candidate gene and assessed endogenous FerLCH expression in the mosquito midgut at 24 hPBM. FerLCH expression was significantly reduced only in the midguts from the mosquitoes injected with dsRNA against AAEL000471 (Fig 4B), in which the level of FerLCH was reduced to about half of that of control (dsEGFP). qRT-PCR on target genes showed that the silencing was effective at the same time point, though the levels were variable, and expression was very low for AAEL012109 (S1 Fig).

#### Effects of gene silencing on the mosquito’s reproductive physiology

**Fig 5:**
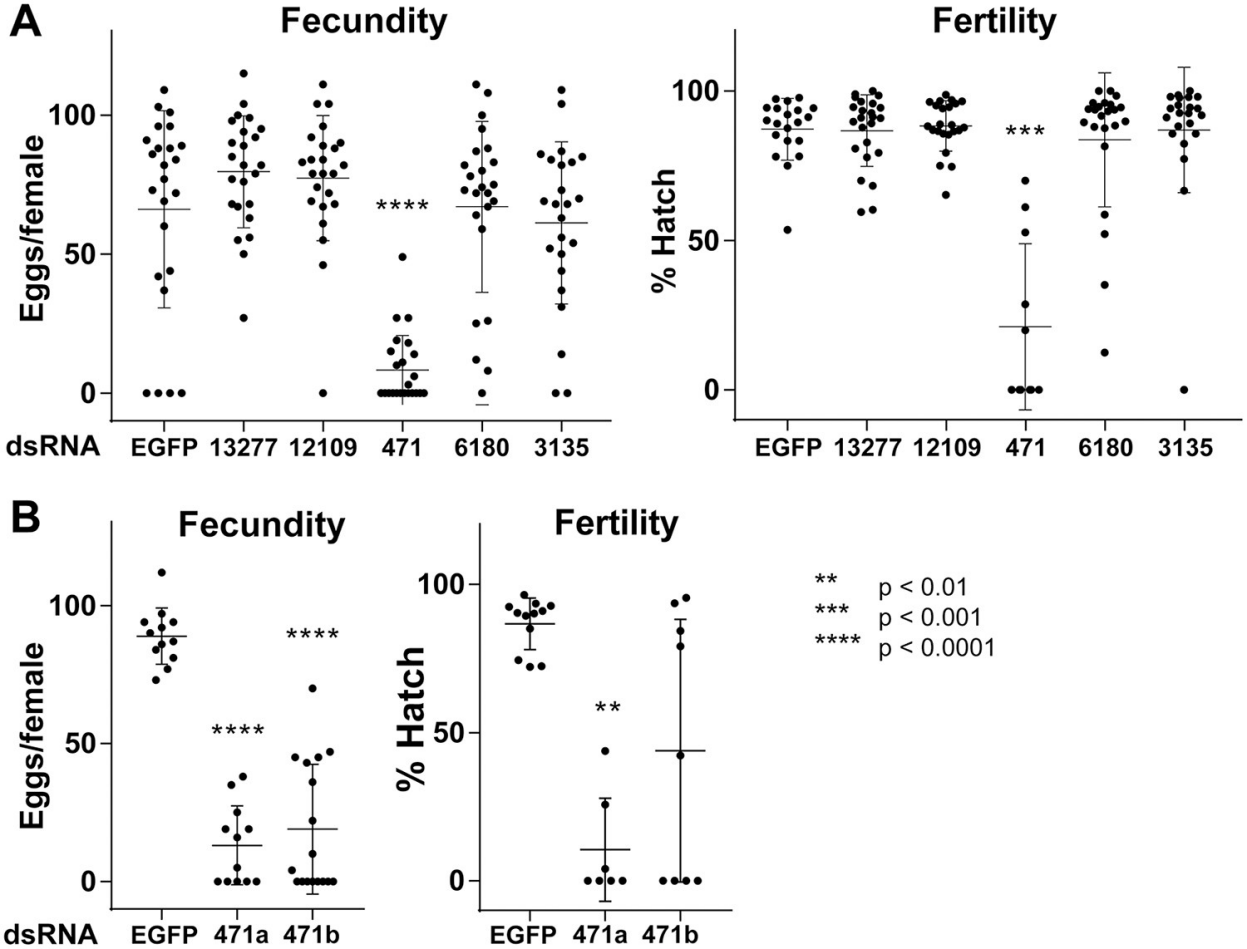
Fecundity and fertility of candidate iron transporter RNAi. (A) Egg number (Fecundity) and hatch rate (Fertility) for female mosquitoes injected with dsRNA against each candidate iron transporter gene. Results are representative of three independent experiments (n = 24). (B) Second dsRNA against AAEL000471 (471b) showed comparable results to the first dsRNA (471a) (n = 12–17). Lines in the graphs show mean ± SD. ANOVA with multiple comparison between EGFP control and each sample was performed for statistical analysis as significant levels shown in the figure.

**Fig 6:**
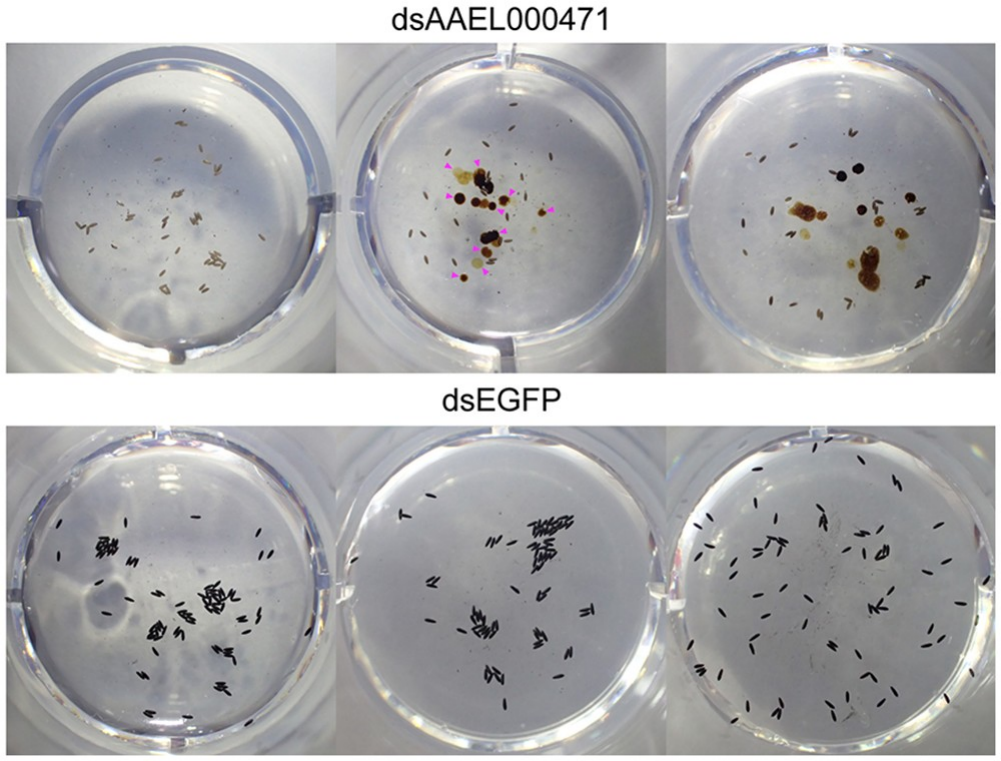
AAEL000471-silenced mosquitoes exhibited small, pale-colored eggs and delayed excretion. Representative EAgaL assay wells after individual females were placed in each well for 48 h (72–120 hPBM). Females injected with dsRNA against AAEL000471 laid small (short), pale-colored eggs and exhibited delayed excretion (magenta arrowheads in the top middle panel).

As bloodmeal iron is thought to be necessary for viable egg production [44,45], we next addressed the importance of each candidate gene in the mosquito’s reproductive fitness following treatment with each dsRNA. Once again, only silencing of AAEL000471 resulted in a significant reduction in fecundity and fertility (Fig 5A). To verify that these effects were specific for AAEL000471, and not due to an unpredicted off-target effect, dsRNA targeting a different region of AAEL000471 was generated, with subsequent silencing showing comparable results (Fig 5B). In addition to defects in both fecundity and fertility, the AAEL000471-silenced females laid small, pale-colored eggs and excreted in the assay wells, indicated delayed excretion (expelling bloodmeal waste and metabolites produced during bloomeal digestion from the alimentary system) (Fig 6). In fact, we observed that a majority of AAEL000471-silenced females still contained blood in the midgut at 72 hPBM, a time when digestion is typically completed. As reduction of AAEL000471 appeared to slow digestion/excretion, we refer to this gene as *dyspepsia* (*dysp*).

#### *Dyspepsia* (*dysp*) is primarily expressed in mosquito Malpighian tubules

**Fig 7:**
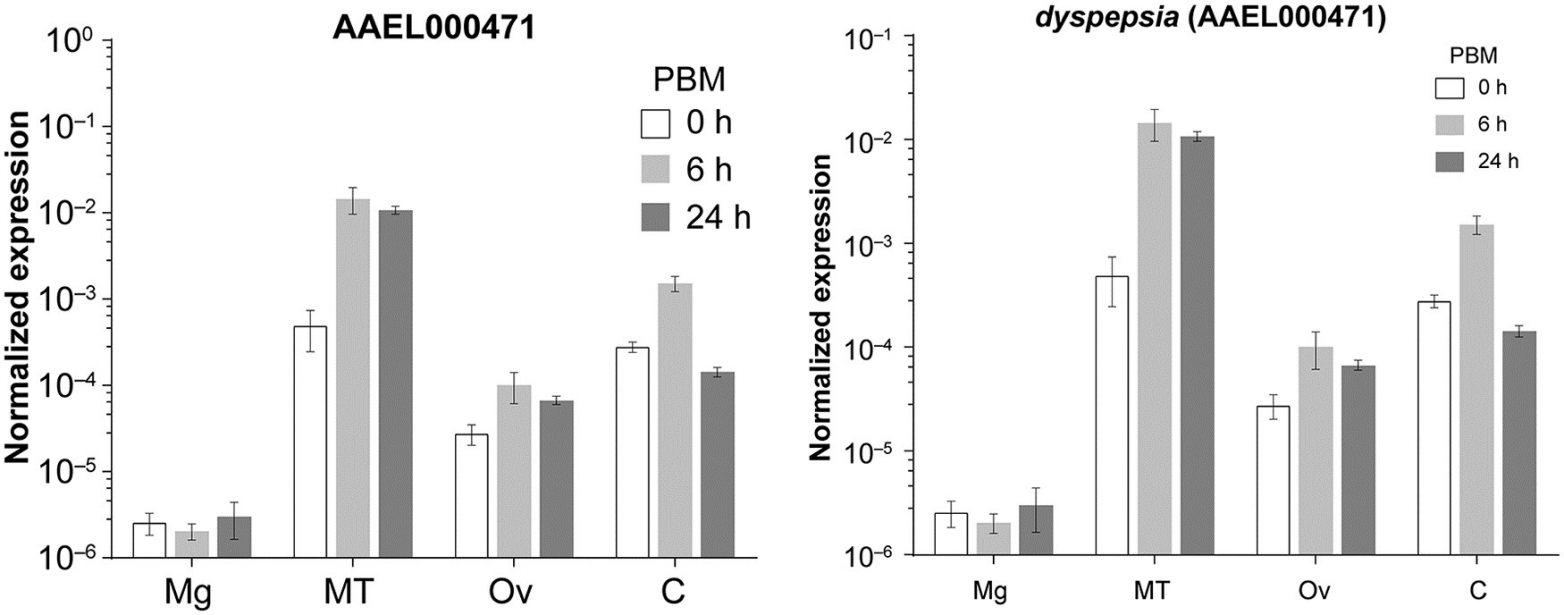
Organ-specific transcript expression of *dysp* (AAEL000471) pre- and post-bloodfeeding. qRT-PCR quantification of mRNA expression in the midgut (Mg), Malpighian tubules (MT), Ovaries (Ov) and remaining body parts (C). Transcript abundance is normalized to the housekeeping gene (rpS7). PBM: post-bloodmeal. The graph shows mean ± SD.

In order to determine where *dysp* might be mediating its physiological effects, we investigated its transcript abundance at pre- and post-bloodfeeding by qRT-PCR. *Dysp* expression was found to be highest in the Malpighian tubules (MT) and lowest in the midgut among the isolated organs. Upon bloodfeeding, *dysp* expression increased most prominently in the MT; about 30 fold and 22 fold at 6 h and 24 hPBM in comparison to the unfed (0 hPBM) state, respectively (Fig 7). Following the MT, remaining body parts (without Mg, MT and Ov: C) and ovaries expressed *dysp* abundantly with an upregulation pattern similar to the MT, albeit to a lesser extent. “C” contains a complex mixture of organs, such as brain, nerve tissues, fat bodies, flight muscle and epidermis, and the specific localization of *dysp* within these tissues remains to be determined.

#### Sequence and phylogenetic analysis of *dyspepsia*

**Fig 8:**
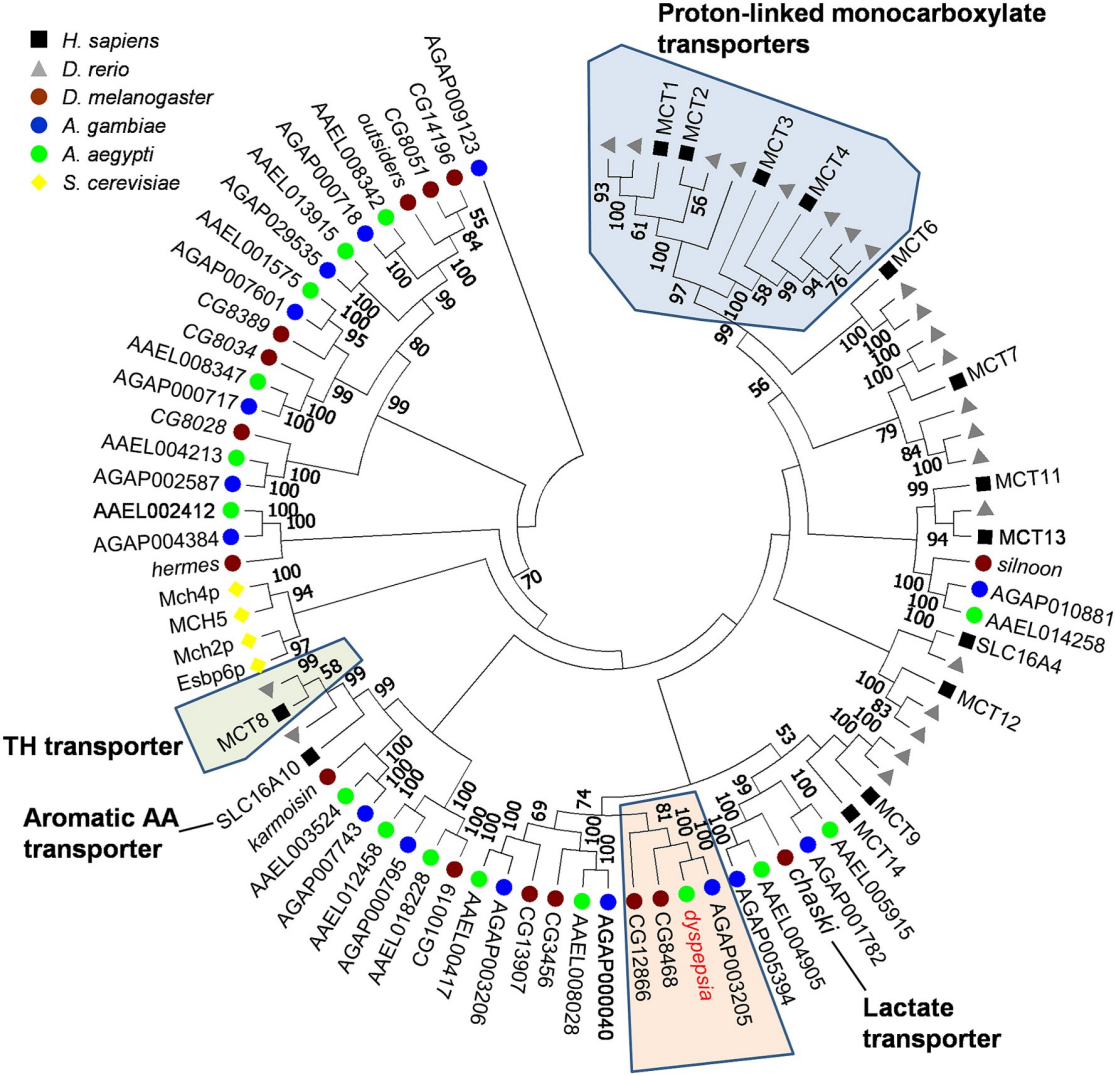
Phylogenetic analysis of SLC16 proteins between vertebrates, dipterans and yeast. Predicted amino acid sequences of 86 SLC16 members were aligned using Muscle as implemented in MEGA7 under the default parameters. The resulting alignment was used to generate a tree using the Neighbor-joining method, with the pairwise deletion option and bootstrapping (n = 1000). The bootstrap consensus tree is displayed, with percent bootstrap support indicated on each node when greater than 50%. SLC16 members with known substrates are indicated; dipteran-specific clade containing *dysp* is highlighted orange.

*Dysp* is predicted to encode a 689 amino-acid polypeptide with 12 predicted transmembrane domains (TMD) [27], and a long cytoplasmic loop between TMD 6 and 7 (S2 Fig), which shows sequence similarity to the solute carrier 16 (SLC16) transporter family. Alignment with human SLC16 proteins indicated that the long cytoplasmic loop is only present in *dysp* (S3 Fig). To better establish the relationship of *dysp* with the SLC16 proteins, only some of whom have characterized substrates, we performed a phylogenetic comparison between homologs identified in dipterans *D*. *melanogaster* (fruit fly) and *An*. *gambiae* (African malaria mosquito), as well as the zebrafish *D*. *rerio* and the budding yeast *S*. *cerevisiae* (Fig 8). Most vertebrate SLC16 genes clustered in the distal clades, whereas dipteran SCL16 genes clustered in the basal clades. This suggests that most of the dipteran SLC16 genes could have evolved independently from their vertebrate counterparts, presumably for specific functions. *dysp* was found to cluster together with a single ortholog in *An*. *gambiae (*AGAP003205*)* with 100% bootstrap support. These two genes group with 100% support with CG8468, indicating this gene as the likely fly ortholog. A second fly gene, CG12866, clustered outside this group with 81% support. Alignment of *dysp* with mosquito and *D*. *melanogaster* genes showed high amino acid identity within the mosquito orthologs implying the potential for conserved functions (S4 Fig). No functional study has been conducted on any of its orthologs, but a separate homolog in *Drosophila melanogaster* (CG3409, *chaski*) has been characterized to be a lactate/pyruvate transporter [46].

## Discussion

Although studies have indicated that iron is required for viable egg production in *Aedes* mosquitoes [44,45], mechanisms for transportation and handling of iron are not well understood with an exception of some parts of the ferritin pathway. In this study, we performed a screen to identify additional genes critical for iron transport in *Ae*. *aegypti*, analyzing genes expressed in an *Ae*. *aegypti* cell line (Aag2) under different iron-stress by RNAseq. Differences in medium iron content caused a significant change in gene expression in Aag2 cells, and we selected 21 genes as iron transporter candidates (Table 1). These RNAseq data may also be useful for the study of iron-generated responses in *Ae*. *aegypti*, such as antioxidation, iron-detoxification, damage-response to reactive oxygen species, immunity and iron (or metal)-responsive *cis*-regulatory motifs for the differentially expressed genes, however in this work we focused on finding iron transporters.

To confirm a role in iron homeostasis, we individually silenced each gene in cells and assessed the cytosolic iron levels by luciferase reporter expression. We had previously used a two-plasmid system to measure iron levels [20]. However, co-transfection results in subpopulations that take up only one of the two plasmids, and imprecision in the ratios of the two plasmids results in greater variation in the output data. The new one-plasmid system we used in this study eliminated these concerns, increasing sensitivity and decreasing variation while also serving to simplify the protocol. Twelve out of the 21 candidate genes showed reduced luciferase expression as compared to controls, despite the fact that at least one had been predicted to act as an exporter from its transcriptional responses to iron, even though a positive control (AaeZIP13) clearly showed increased luciferase expression (Fig 3). This might be due to pH dependency or a requirement for co-transporting or exchanger molecules. Moreover, we could not exclude the involvement of other metal ions such as zinc and cadmium, which in *D*. *melanogaster* increased ferritin expression [47,48]. We previously demonstrated that the *Ae*. *aegypti* FerLCH promoter was ~50-fold less sensitive to Cu^2+^ and could not be activated by Zn^2+^ in Aag2 cells [20]. However, in *D*. *melanogaster* ferritin expression could be induced upon zinc supplementation in some cell types distinct from those that responded to iron [48], implying cell-type dependent promoter responsiveness may also exist in *Ae aegypti*. We selected five genes that showed the most prominent difference (more than 0.8log_2_-fold) from the control for downstream *in vivo* analysis.

To investigate iron transporting function of the selected genes *in vivo*, we quantified FerLCH expression in the midgut of gene-silenced mosquitoes in comparison to control (dsEGFP). Ferritin expression has previously been shown to be elevated after bloodmeal [42,43,49,50]. FerLCH expression was highly upregulated in the midgut upon bloodmeal, which was significantly reduced only in *dysp* (AAEL000471)-silenced mosquitoes. This implies that *dysp* may indeed possess an iron-transporter function. However, *dysp* showed very low expression in the midgut (Fig 8), and the reduction of FerLCH in *dysp*-silenced mosquito’s midgut could also be explained by a general reduction of digestion due to the systemic effects of impaired Malpighian tubules (MT) function. Hence, the state of *dysp* as a true iron transporter is inconclusive, and additional investigations are needed to address the cause of FerLCH reduction in the midgut. *dysp*-silenced mosquitoes also showed a significant reduction of both fecundity and fertility (Fig 5) and signs of delayed excretion (Fig 6). These results indicate the importance of *dysp* in the mosquito’s reproductive fitness and excretion/digestion. Moreover, pale-colored eggs produced by *dysp*-silenced mosquitoes suggest defects in the sclerotization process. Enzymes involved in cuticle tanning or sclerotization include heme-containing peroxidases, copper-containing tyrosinase, laccase and phenoloxidases [51], potentially implicating *dysp* in copper homeostasis as well as iron. Nonetheless, as in the case of FerLCH expression, a direct link between reproductive fitness and iron transport function of *dysp* remains to be confirmed.

Organ-specific expression analysis showed that *dysp* was highly enriched in the MT, which are equivalent to the vertebrate kidney, suggesting a potential role in excretion. Substantial upregulation of *dysp* in the MT after bloodmeal also supports a role in excretion related to bloodmeal digestion (Fig 8). We previously found that AaeZIP11 and AaeZnT7 displayed elevated expression at 24 hPBM and 6 hPBM, respectively in the MT suggesting a role in bloodmeal digestion and excretion [20]. The 1:1 ortholog of *dysp* in *An*. *gambiae* (AGAP003205) was also found to be enriched in the MT [52] and is upregulated upon bloodfeeding [53], while the *D*. *melanogaster* ortholog (CG8468), but not another homolog (CG12866) is highly enriched in the MT [54]. Together, this suggests a conserved function for *dsyp* orthologs in excretion in Diptera. High amino acid sequence conservation within the mosquito orthologs (S4 Fig) further suggests conserved (in mosquitoes) and derived (from other dipterans) function, perhaps related to bloodmeal digestion.

As the excretory organ, a major focus in the research on mosquito MT is ion transport systems, which play crucial roles upon bloodfeeding to remove excess ions (mainly K^+^, Na^+^ and Cl^–^) and water [55]. For instance, an inhibitor of Kir (inward rectifier K^+^) channels impaired urine production and reduced fecundity in *An*. *gambiae* and *Ae*. *aegypti* [56]. Another Kir inhibitor also impaired excretion and caused incapacitation and mortality in *Ae*. *aegypti* [57]. Silencing of aquaporin water channels also caused a similar excretion deficiency in *Ae*. *aegypti* [58]. MT are also suggested to play a key role in detoxification [59]. However, detoxification as well as transporting toxic materials including iron by the MT has not been well documented. An aforementioned iron transporter, AnaNRAMP is expressed abundantly in MT [17], which seemed to be absent in culicine mosquitoes [20].

Moderate expression of *dysp* was also detected in the ovaries and remaining body parts. In the *Drosophila* ortholog (CG8468) expression is high in the fat bodies, implying that *dysp* expression found in other tissues (Fig 7C) could represent the fat bodies and might explain its expression in the organs important for nutrient (including iron) processing and transport.

*dysp* has sequence similarity to SLC16 family transporters. Described SLC16 members transport monocarboxylates (SLC16A1, 3, 7, 8), thyroid hormones (SLC16A2, 10), aromatic amino acids (SLC16A10) and the drug bumetanide (SLC16A5), but substrates of a majority of homologues are unknown [60]. Alignments suggest that vertebrate and dipteran SLC16 genes may have different functions as vertebrate and dipteran members have undergone independent expansions. Thus, the substrates and physiological roles may be substantially different. Altogether, further studies on its real substrates, potential inhibitors and its importance in the mosquito’s reproduction, excretion and digestion should benefit development of novel mosquito control.

## Supporting information

S1 Fig qRT-PCR analysis of transcript reduction of the target genes. The graphs show the normalized expression (to rpS7) in the midguts of the mosquitoes used to assess FerLCH expression (Fig 4B). Numbers on the x axis indicate the AAEL numbers whose gene expression assessed for each panel where dark grey bars represent dsEGFP-injected control and light grey bar represents dsTarget-injected treatments.
(TIF)

S2 Fig Graphical presentation of membrane topology predicted by TOPCONS. Orange band represents phospholipid membrane; blue numbers indicate transmembrane domains. “extra”: extracellular; “intra”: intracellular.
(TIF)

S3 Fig Alignment of AAEL000471 with human SLC16 homologs and percent identity matrix. (PDF)

S4 Fig Alignment of AAEL000471 with selected mosquito orthologs and *D*. *melanogaster* ortholog and percent identity matrix. (PDF)

S1 File List of primers used for dsRNA template amplification and qRT-PCR with determined E values and GenBank accession numbers/VectorBase geneIDs for the amino acid sequences used for gene tree construction and alignment. (XLSX)

S2 File Original differential gene expression table for AaegL3 and AaegL5 mapped results. (XLSX)

S3 File Normalized count tables for Aag2 and A20 cell lines mapped to AaegL5 genome. (XLSX)
