# Supplementary figures and images for: Aedes aegypti dyspepsia encodes a novel member of the SLC16 family of transporters and is critical for reproductive fitness
Source: PLoS Negl Trop Dis. 2021 Apr 7;15(4):e0009334. doi: 10.1371/journal.pntd.0009334 (PMC8055033; doi:10.1371/journal.pntd.0009334)

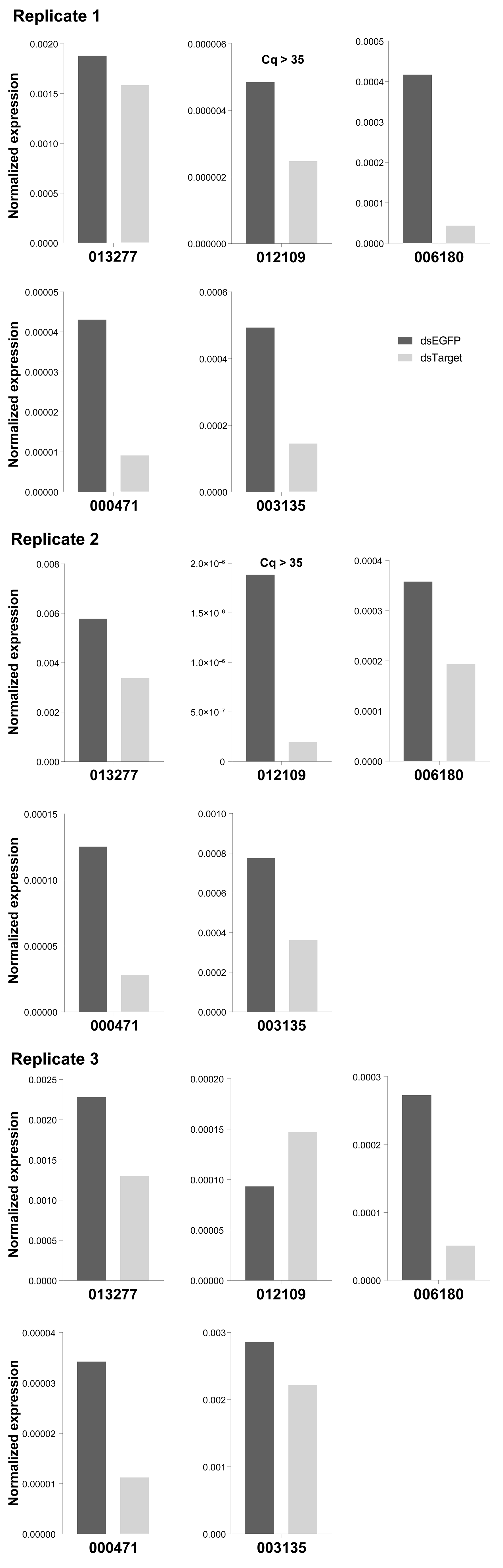

Supplement: S1 Fig — The graphs show the normalized expression (to rpS7) in the midguts of the mosquitoes used to assess FerLCH expression (Fig 4B). Numbers on the x axis indicate the AAEL numbers whose gene expression assessed for each panel where dark grey bars represent dsEGFP-injected control and light grey bar represents dsTarget-injected treatments. (TIF) [file pntd.0009334.s001.tif]

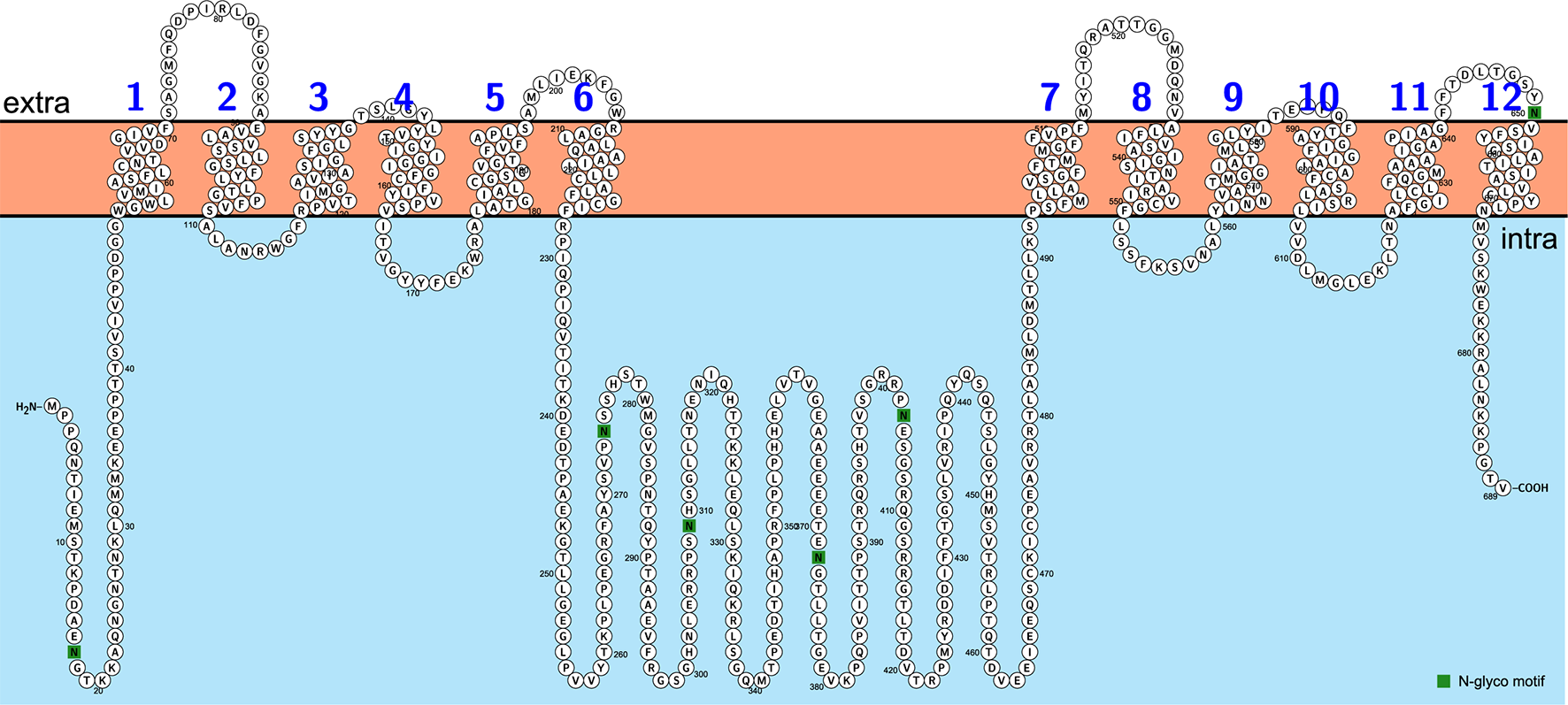

Supplement: S2 Fig — Orange band represents phospholipid membrane; blue numbers indicate transmembrane domains. “extra”: extracellular; “intra”: intracellular. (TIF) [file pntd.0009334.s002.tif]
