# Supplementary material for: Aedes aegypti dyspepsia encodes a novel member of the SLC16 family of transporters and is critical for reproductive fitness
Source: PLoS Negl Trop Dis. 2021 Apr 7;15(4):e0009334. doi: 10.1371/journal.pntd.0009334 (PMC8055033; doi:10.1371/journal.pntd.0009334)
Supplement: S3 Fig — (PDF) [file pntd.0009334.s003.pdf]

Transmembrane domains predicted by TOPCONS

Cytoplasmic loop of AAEL000471

```
SLC16A1         ------------------------------------------------------------     0
SLC16A2         MALQSQASEEAKGPWQEADQEQQEPVGSPEPESEPEPEPEPEPVPVPPPEPQPEPQPLPD     60
SLC16A3         ------------------------------------------------------------     0
SLC16A4         ------------------------------------------------------------     0
SLC16A5         ------------------------------------------------------------     0
SLC16A6         ------------------------------------------------------------     0
SLC16A7         ------------------------------------------------------------     0
SLC16A8         ------------------------------------------------------------     0
SLC16A9         ------------------------------------------------------------     0
SLC16A10        MVLSQEEPDSARGT------SEAQPLGP------------------APT--GAAPPPGPG     34
SLC16A11        ------------------------------------------------------------     0
SLC16A12        ----------------------------------------------MPSGSHWTA-----     9
SLC16A13        ------------------------------------------------------------     0
SLC16A14        -----------------------------------------------------------M     1
AAEL000471      ----------------------------------------------MPPQNTIEM-----     9


SLC16A1         -------------------------MPPAVGGPVGYTPPDGGWGWAVVIGAFISIGFSYA     35
SLC16A2         PAPLPELEFESERVHEPE----PTPTVETRGTARGFQPPEGGFGWVVVFAATWCNGSIFG     116
SLC16A3         -----------------------MGGAVVDEGPTGVKAPDGGWGWAVLFGCFVITGFSYA     37
SLC16A4         ------------------------MLKREGKVQPYTKTLDGGWGWMIVIHFFLVNVFVMG     36
SLC16A5         -------------------------------MPQALERADGSWAWVVLLATMVTQGLTLG     29
SLC16A6         --------------------MTQNKLKLCSKANVYTEVPDGGWGWAVAVSFFFVEVFTYG     40
SLC16A7         -------------------------MPPMPSAPPVHPPPDGGWGWIVVGAAFISIGFSYA     35
SLC16A8         -----------------------MGA---GGPRRGEGPPDGGWGWVVLGACFVVTGFAYG     34
SLC16A9         --------------------------------MELKKSPDGGWGWVIVFVSFLTQFLCYG     28
SLC16A10        PSDSPEAAVEKVEVELAG----P----ATAEPHEPPEPPEGGWGWLVMLAAMWCNGSVFG     86
SLC16A11        ------------------------------MTPQPAGPPDGGWGWVVAAAAFAINGLSYG     30
SLC16A12        -NSSKI--ITWL-LEQPGK-EEKRKTMAKVNRARSTSPPDGGWGWMIVAGCFLVTICTRA     64
SLC16A13        -------------------------------MARRTEPPDGGWGWVVVLSAFFQSALVFG     29
SLC16A14        YTSHED--IGYD----------FEDGPKDKKTLKPHPNIDGGWAWMMVLSSFFVHILIMG     49
AAEL000471      -STKPDAENGTK-KAQNGNTNKLQMMKEEPPTTSVIVPPDGGWGWLVMIASFLCNTVVDG     67
                                                       :*.:.* :            .

SLC16A1         FPKSITVFFKEIEGIFHATT-----SEVSWISSIMLAVMYGGGPISSILVNKYGSRIVMI     90
SLC16A2         IHNSVGILYSMLLEEEK-EKNRQVEFQAAWVGALAMGMIFFCSPIVSIFTDRLGCRITAT     175
SLC16A3         FPKAVSVFFKELIQEFGIGY-----SDTAWISSILLAMLYGTGPLCSVCVNRFGCRPVML     92
SLC16A4         MTKTFAIFFVVFQEEFEGTS-----EQIGWIGSIMSSLRFCAGPLVAIICDILGEKTTSI     91
SLC16A5         FPTCIGIFFTELQWEFQASN-----SETSWFPSILTAVLHMAGPLCSILVGRFGCRVTVM     84
SLC16A6         IIKTFGVFFNDLMDSFNESN-----SRISWIISICVFVLTFSAPLATVLSNRFGHRLVVM     95
SLC16A7         FPKAVTVFFKEIQQIFHTTY-----SEIAWISSIMLAVMYAGGPVSSVLVNKYGSRPVVI     90
SLC16A8         FPKAVSVFFRALMRDFDAGY-----SDTAWVSSIMLAMLYGTGPVSSILVTRFGCRPVML     89
SLC16A9         SPLAVGVLYIEWLDAFGEGK-----GKTAWVGSLASGVGLLASPVCSLCVSSFGARPVTI     83
SLC16A10        IQNACGVLFVSMLETFGSKDDDKMVFKTAWVGSLSMGMIFFCCPIVSVFTDLFGCRKTAV     146
SLC16A11        LLRSLGLAFPDLAEHFDRSA-----QDTAWISALALAVQQAASPVGSALSTRWGARPVVM     85
SLC16A12        VTRCISIFFVEFQTYFTQDY-----AQTAWIHSIVDCVTMLCAPLGSVVSNHLSCQVGIM     119
SLC16A13        VLRSFGVFFVEFVAAFEEQA-----ARVSWIASIGIAVQQFGSPVGSALSTKFGPRPVVM     84
SLC16A14        SQMALGVLNVEWLEEFHQSR-----GLTAWVSSLSMGITLIVGPFIGLFINTCGCRQTAI     104
AAEL000471      IVFSAGMFQDPIRLDFGVGK-----AEVALVSSLLSGFYLLTGPFVSALANRWGFRPVTI     122
                      :                     . . ::   .     *.        . :

SLC16A1         VGGCLSGCGLIAASFCNTVQQLYVCIGVIGGLGLAFNLNPALTMIGKYFYKRRPLANGLA     150
SLC16A2         AGAAVAFIGLHTSSFTSSLSLRYFTYGILFGCGCSFAFQPSLVILGHYFQRRLGLANGVV     235
SLC16A3         VGGLFASLGMVAASFCRSIIQVYLTTGVITGLGLALNFQPSLIMLNRYFSKRRPMANGLA     152
SLC16A4         LGAFVVTGGYLISSWATSIPFLCVTMGLLPGLGSAFLYQVAAVVTTKYFKKRLALSTAIA     151
SLC16A5         LGGVLASLGMVASSFSHNLSQLYFTAGFITGLGMCFSFQSSITVLGFYFVRRRVLANALA     144
SLC16A6         LGGLLVSTGMVAASFSQEVSHMYVAIGIISGLGYCFSFLPTVTILSQYFGKRRSIVTAVA     155
SLC16A7         AGGLLCCLGMVLASFSSSVVQLYLTMGFITGLGLAFNLQPALTIIGKYFYRKRPMANGLA     150
SLC16A8         AGGLLASAGMILASFATRLLELYLTAGVLTGLGLALNFQPSLIMLGLYFERRRPLANGLA     149
SLC16A9         FSGFMVAGGLMLSSFAPNIYFLFFSYGIVVGLGCGLLYTATVTITCQYFDDRRGLALGLI     143
SLC16A10        VGAAVGFVGLMSSSFVSSIEPLYLTYGIIFACGCSFAYQPSLVILGHYFKKRLGLVNGIV     206
SLC16A11        VGGVLASLGFVFSAFASDLLHLYLGLGLLAGFGWALVFAPALGTLSRYFSRRRVLAVGLA     145
SLC16A12        LGGLLASTGLILSSFATSLKHLYLTLGVLTGLGFALCYSPAIAMVGKYFSRRKALAYGIA     179
SLC16A13        TGGILAALGMLLASFATSLTHLYLSIGLLSGSGWALTFAPTLACLSCYFSRRRSLATGLA     144
SLC16A14        IGGLVNSLGWVLSAYAANVHYLFITFGVAAGLGSGMAYLPAVVMVGRYFQKRRALAQGLS     164
AAEL000471      MGAVIASIGFGLSYYGTSLGYLYVTYGIIGGIGFCFIYVPSVITVGYYFEKWRALATGIA     182
                 .. .   *   : :   :    .  *.  . *  :    :      **     :  .:
```

```
SLC16A1      MAGSPVFLCTLAPLNQVFFGIFGWRGSFLILGGLLLNCCVAGALMRPIGPKPTKAGKDKS   210
SLC16A2      SAGSSIFSMSFPFLIRMLGDKIKLAQTFQVLSTFMFVLMLLSLTYRPLLPSSQDTP----   291
SLC16A3      AAGSPVFLCALSPLGQLLQDRYGWRGGFLILGGLLLNCCVCAALMRPLVVTAQ-------   205
SLC16A4      RSGMGL-TFLLAPFTKFLIDLYDWTGALILFGAIALNLVPSSMLLRPIHIKSENNSGIKD   210
SLC16A5      SMGVSLGITLWPLLSRYLLENLGWRGTFLVFGGIFLHCCICGAIIRPVATSVAPETKECP   204
SLC16A6      STGECFAVFAFAPAIMALKERIGWRYSLLFVGLLQLNIVIFGALLRPIFIRGPASPKIVI   215
SLC16A7      MAGSPVFLSSLAPFNQYLFNTFGWKGSFLILGSLLLNACVAGSLMRPLGPNQTTSKSKNK   210
SLC16A8      AAGSPVFLSALSPLGQQLLERFGWRGGFLLLGGLLLHCCACGAVMRPPPGPGPRPRRDSA   209
SLC16A9      STGSSVGLFIYAALQRMLVEFYGLDGCLLIVGALALNILACGSLMRPLQSSDCPLPKKI-   202
SLC16A10     TAGSSVFTILLPLLLRVLIDSVGLFYTLRVLCIFMFVLFLAGFTYRPLATSTKDKE----   262
SLC16A11     LTGNGASSLLLAPALQLLLDTFGWRGALLLLGAITLHLTPCGALLLPLVLPGDPPA----   201
SLC16A12     MSGSGIGTFILAPVVQLLIEQFSWRGALLILGGFVLNLCVCGALMRPITLKEDHTTPEQN   239
SLC16A13     LTGVGLSSFTFAPFFQWLLSHYAWRGSLLLVSALSLHLVACGALLRPPSLAEDPAV----   200
SLC16A14     TTGTGFGTFLMTVLLKYLCAEYGWRNAMLIQGAVSLNLCVCGALMRPLSPGKNPNDPGEK   224
AAEL000471   LCGSGVGTFVFAPLSAMLIEKFGWRGALLAQAAIILLCALFGCIFRPIQPIQVTITKDED   242
               *              :         :     . :     .    *

SLC16A1      KASLEK--------------------------------------AG--------------   218
SLC16A2      --SKR-------------------------------------------------------   294
SLC16A3      ------------------------------------------------------------   205
SLC16A4      KGSSLS--------------------------------------AHGPEAHA--------   224
SLC16A5      PPPPET------------------------------------------------------   210
SLC16A6      QENRKE--------------------------------------AQYM------------   225
SLC16A7      TGKTE-------------------------------------------------------   215
SLC16A8      G---DR--------------------------------------AG--------------   214
SLC16A9      APEDLP--------------------------------------DKYS----IYNEKGKN   220
SLC16A10     --SGGS------------------------------------------------------   266
SLC16A11     ------------------------------------------------------------   201
SLC16A12     HVCRTQ--------------------------------------KE--------------   247
SLC16A13     ------------------------------------------------------------   200
SLC16A14     DVRGLP--------------------------------------AHSTESVKSTGQQGRT   246
AAEL000471   TPAEKGTLLGEGLPVVYTKPLPEGRFAYSVPNSSHSTWMGVSPNTQYPTAAEVFRGSGHN   302


SLC16A1      ------------------------------------------------------------   218
SLC16A2      ------------------------------------------------------------   294
SLC16A3      ------------------------------------------------------------   205
SLC16A4      --------TETHCHETEEST---------I----------KDST----------------   241
SLC16A5      ------------------------------------------------------------   210
SLC16A6      -----------LENEKTRTS---------I-----------DSI----------------   238
SLC16A7      ------------------------------------------------------------   215
SLC16A8      ------------------------------------------------------------   214
SLC16A9      LEENINILDKSYS----------SEEKCRITL----------------------------   242
SLC16A10     ------------------------------------------------------------   266
SLC16A11     ------------------------------------------------------------   201
SLC16A12     ------------------------------------------------------------   247
SLC16A13     ------------------------------------------------------------   200
SLC16A14     EEKDGGLG----N----------EETLCDLQA----------------------------   264
AAEL000471   LERRPSNHSGLLTNENIQHTTKKLEQLSKIQKRLSGQMTPEDTIHAPRFPLPHHELVTVG   362


SLC16A1      --------KSG----------------------------------------------VKK   224
SLC16A2      ----------G-----------------------------------------------VR   297
SLC16A3      ------------------------------------------------------------   205
SLC16A4      --------TQKAGLPSK---------N------------------------------LTV   254
SLC16A5      ------------------------------------------------------------   210
SLC16A6      --------DSGVELTTS---------P------------------------------KNV   251
SLC16A7      ------------------------------------------------------------   215
SLC16A8      --------DAP----------------------------------------------GEA   220
SLC16A9      -------------ANGDWKQDS------LLHKNPTVTHTKEPETYKKKVAEQ--------   275
SLC16A10     ----------G-----------------------------------------------SS   269
SLC16A11     ------------------------------------------------------------   201
SLC16A12     --------DIK----------------------------------------------RVS   253
SLC16A13     ------------------------------------------------------------   200
SLC16A14     ------------------------------QECPDQAGHRKNMCALRILKTV--------   286
AAEL000471   EAAEEEETENGTLLTGEVKPQPVITTPSTRQRSHTVSGRRPNESGSRQGSRRGTLTDVTR   422
```

```
SLC16A1         DLHDA----NT--------------------------DLIGRHPKQ----EKRSVFQTIN    250
SLC16A2         T-------------------------------------------------LHQRFLAQLR    308
SLC16A3         --------------------------------------------------PGSGPPRPSR    215
SLC16A4         SQNQSEEFYNG--------------------------PNRNRLLLKSDEESDKVISWSCK    288
SLC16A5         P------------------------------------------ALG----CLAACGRTIQ    224
SLC16A6         PTHTNLELEPK--------------------------ADMQQVLVK----TSPRPSEKKA    281
SLC16A7         -------------------------------------DDSSPKKIK----TKKSTWEKVN    234
SLC16A8         EA-----------------------------------DG-AGLQLR----EASPRVRPRR    240
SLC16A9         ------TY--------------------------------------FCKQLAKRKWQLYK    291
SLC16A10        L-------------------------------------------------FSRKKFSPPK    280
SLC16A11        -----------------------------------------------------P-PRSPL    207
SLC16A12        PY--------S--------------------------SLTKEWAQT----CLCCCLQQE-    274
SLC16A13        -----------------------------------------------------GGPRAQ-    206
SLC16A14        ------SW--------------------------------------LTMRVRKGFEDWYS    302
AAEL000471      PMYRDDIFFTGSLVRIPQYQSQTSLGYHMSVTRLPTQTDVEEIEEQSCKICPEAVRRTLA    482

SLC16A1         QFL--DLTLF-THRGFLLYL-SGNVIMFFGLFAPLVFLSSYGKSQHYSSE-KSAFLLSIL    305
SLC16A2         KYF--NMRVF-RQRTYRIWA-FGIAAAALGYFVPYVHLMKYVEEEFSEIK-ETWVLLVCI    363
SLC16A3         RLL--DLSVF-RDRGFVLYA-VAASVMVLGLFVPPVFVVSYAKDLGVPDT-KAAFLLTIL    270
SLC16A4         QLF--DISLF-RNPFFYIFT-WSFLLSQLAYFIPTFHLVARAKTLGIDIM-DASYLVSVA    343
SLC16A5         RHL--AFDILRHNTGYCVYI-LGVMWSVLGFPLPQVFLVPYAMWHSVDEQ-QAALLISII    280
SLC16A6         PLL--DFSIL-KEKSFICYA-LFGLFATLGFFAPSLYIIPLGISLGIDQD-RAAFLLSTM    336
SLC16A7         KYL--DFSLF-KHRGFLIYL-SGNVIMFLGFFAPIIFLAPYAKDQGIDEY-SAAFLLSVM    289
SLC16A8         RLL--DLAVC-TDRAFAVYA-VTKFLMALGLFVPAILLVNYAKDAGVPDT-DAAFLLSIV    295
SLC16A9         NYCGETVALF-KNKVFSALFIAILLFDIGG-FPPSLLMEDVARSSNVKEEEFIMPLISII    349
SLC16A10        KIF--NFAIF-KVTAYAVWA-VGIPLALFGYFVPYVHLMKHVNERFQDEK-NKEVVLMCI    335
SLC16A11        AAL--GLSLF-TRRAFSIFA-LGTALVGGGYFVPYVHLAPHALDRGLGGY-GAALVVAVA    262
SLC16A12        ------YSFL-LMSDFVVLA-VSVLFMAYGCSPLFVYLVPYALSVGVSHQ-QAAFLMSIL    325
SLC16A13        -----LTSLL-HHGPFLRYT-VALTLINTGYFIPYLHLVAHLQDLDWDPL-PAAFLLSVV    258
SLC16A14        GYFGT-ASLF-TNRMFVAFIFWA-LFAYSSFVIPFIHLPEIVNLYNLSEQNDVFPLTSII    359
AAEL000471      TML--DMTLL-KSPSFMLLA-VSGFFTMMGFFVPFMYITQRATTGGMDQN-VALFIVSAI    537
                        .      :             .     . :                 :

SLC16A1         AFVDMVARPSMGLVANTKPIRPRIQY-F----FAASVVANGVCHMLAPLSTTYVGFCVYA    360
SLC16A2         GATSGLGRLVSGHISDSIPGLKKIYLQV------LSFLLLGLMSMMIPLCRDFGGLIVVC    417
SLC16A3         GFIDIFARPAAGFVAGLGKVRPYSVY-L----FSFSMFFNGLADLAGSTAGDYGGLVVFC    325
SLC16A4         GILETVSQIISGWVADQNWIKKYHYHKS-------YLILCGITNLLAPLATTFPLLMTYT    396
SLC16A5         GFSNIFLRPLAGLMAGRPAFASHRKY-L----FSLALLLNGLTNLVCAASGDFWVLVGYC    335
SLC16A6         AIAEVFGRIGAGFVLNREPIRKIYIELI-------CVILLTVSLFAFTFATEFWGLMSCS    389
SLC16A7         AFVDMFARPSVGLIANSKYIRPRIQY-F----FSFAIMFNGVCHLLCPLAQDYTSLVLYA    344
SLC16A8         GFVDIVARPACGALAGLARLRPHVPY-L----FSLALLANGLTDLSSARARSYGALVAFC    350
SLC16A9         GIMTAVGKLLLGILADFKWINTLYLYVA-------TLIIMGLALCAIPFAKSYVTLALLS    402
SLC16A10        GVTSGVGRLLFGRIADYVPGVKKVYLQV------LSFFFIGLMSMMIPLCSIFGALIAVC    389
SLC16A11        AMGDAGARLVCGWLADQGWVPLPRLLAVFGALTGLGLWVVGLVPVVGGEESWGGPLLAAA    322
SLC16A12        GVIDIIGNITFGWLTDRRCLKNYQYV-C----YLFAVGMDGLCYLCLPMLQSLPLLVPFS    380
SLC16A13        AISDLVGRVVSGWLGDAVPGPVTRLLMLWTTLTGVSLALFP---VAQ----APTALVALA    311
SLC16A14        AIVHIFGKVILGVIADLPCISVWNVFLL-------ANFTLVLSIFILPLMHTYAGLAVIC    412
AAEL000471      GISNTIARIVCGFLSSFKSVNALYINNV-------AITMGGIATMLSGLYITEAFQFTYA    590
                .      .   * : .

SLC16A1         GFFGFAFGWLSSVLFETL--MDLVGPQRFSSAVGLVTIVECCPVLLGPPLLGRLNDMYGD    418
SLC16A2         LFLGLCDGFFITIMAPIA--FELVGPMQASQAIGYLLGMMALPMIAGPPIAGLLRNCFGD    475
SLC16A3         IFFGISYGMVGALQFEVL--MAIVGTHKFSSAIGLVLLMEAVAVLVGPPSGGKLLDATHV    383
SLC16A4         ICFAIFAGGYLALILPVL--VDLCRNSTVNRFLGLASFFAGMAVLSGPPIAGWLYDYTQT    454
SLC16A5         LAYSVSMSGIGALIFQVL--MDIVPMDQFPRALGLFTVLDGLAFLISPPLAGLLLDATNN    393
SLC16A6         IFFGFMVGTIGGTHIPLLAEDDVVGIEKMSSAAGVYIFIQSIAGLAGPPLAGLLVDQSKI    449
SLC16A7         VFFGLGFGSVSSVLFETL--MDLVGAPRFSSAVGLVTIVECGPVLLGPPLAGKLVDLTGE    402
SLC16A8         VAFGLSYGMVGALQFEVL--MAAVGAPRFPSALGLVLLVEAAAVLIGPPSAGRLVDVLKN    408
SLC16A9         GILGFLTGNWSIFPY-VT--TKTVGIEKLAHAYGILMFFAGLGNSLGPPIVGWFYDWTQT    459
SLC16A10        LIMGLFDGCFISIMAPIA--FELVGAQDVSQAIGFLLGFMSIPMTVGPPIAGLLRDKLGS    447
SLC16A11        VAYGLSAGSYAPLVFGVL--PGLVGVGGVVQATGLVMMLMSLGGLLGPPLSGFLRDETGD    380
SLC16A12        CTFGYFDGAYVTLIPVVT--TEIVGTTSLSSALGVVYFLHAVPYLVSPPIAGRLVDTTGS    438
SLC16A13        VAYGFTSGALAPLAFSVL--PELIGTRRIYCGLGLLQMIESIGGLLGPPLSGYLRDVTGN    369
SLC16A14        ALIGFSSGYFSLMPV-VT--EDLVGIEHLANAYGIIICANGISALLGPPFAGWIYDITQK    469
AAEL000471      GIFGIAIACFSALRSILV--VDLMGLEKLTNAFGILCLFQGMAAAIGAPIAGFFTDLTGS    648
                   .   .                         *            . *  *  : :
```

```
SLC16A1         YKYTYWACGVVLIISGI-YLFIGMGINYRLLAKEQKANEQKKESKEEETSIDVAGKPNEV    477
SLC16A2         YHVAFYFAGVPPIIGAVILFFVPLMHQR-MFKKEQRDSSK-------DKMLAPDPDPNGE    527
SLC16A3         YMYVFILAGAEVLTSSL-ILLLGNFFCIRKKPKE---PQPEVAAAEEEKLH-KPPAD---    435
SLC16A4         YNGSFYFSGICYLLSSVSFFFVPLAERWKNSLT---------------------------    487
SLC16A5         FSYVFYMSSFFLISAAL-FMG-GS--FYALQKKEQGKQAVAADALERDLFLEAK------    443
SLC16A6         YSRAFYSCAAGMALAAV-CLALVRPCKMGLCQHHHSGE-TKVVSHRGKT---LQDIPEDF    504
SLC16A7         YKYMYMSCGAIVVAASV-WLLIGNAINYRLLAKERKEENARQKTRESEPLSKSK-HSEDV    460
SLC16A8         YEIIFYLAGSEVALAGV-FMAVATNCCLRCAKAAPSGPGTEGGASDTEDAE-AEGDSEPL    466
SLC16A9         YDIAFYFSGFCVLLGGFILLLAALPSW-DTCNKQLPKPAP-----------------TTF    501
SLC16A10        YDVAFYLAGVPPLIGGAVLCFIPWIHSK-KQREISKTTGK-------EKMEKMLENQNS-    498
SLC16A11        FTASFLLSGSLILSGSFIYIGLPRALPSCGPASPPATPPPE---T-----GELLPAPQAV    432
SLC16A12        YTAAFLLCGFSMIFSSV-LLGFARLIK-----RMRKTQ-LQFIAKESDPKLQLW------    485
SLC16A13        YTASFVVAGAFLLSGSGILLTLPHFFCFSTTTSGPQDLVTE--AL-----DTKVPLPKEG    422
SLC16A14        YDFSFYICGLLYMIGILFLLIQPCIRIIEQSRRKYMDGA---------------------    508
AAEL000471      YNVSFYISGALITISAVLCYPLNMVSKWEKKRALNKKPGTV-------------------    689
                :   :  ..     .

SLC16A1         TKAAESPDQKDTD------GGPKEEES--------PV-----------------------    500
SLC16A2         -LLPGSPN----P---------EEPI----------------------------------    539
SLC16A3         -----SGVDLREVEHFLKAEPEKNGEVV-----HTPETS---V-----------------    465
SLC16A4         ------------------------------------------------------------    487
SLC16A5         ----DGPGKQRSPEIMCQSSRQPRPAGVNKHLWGCPASSRTSHEWLLWPKAVLQAKQTAL    499
SLC16A6         LEMDLAKNEHR-------VHVQME-----------PV-----------------------    523
SLC16A7         NVKVSN-AQ----------SVTSERET--------NI-----------------------    478
SLC16A8         PVVAEEPGNLEALEVLSARGEPTEPEIE-----ARPRLA---AESV--------------    504
SLC16A9         LYKVASNV----------------------------------------------------    509
SLC16A10        -LLSSSSGMFKKE---------SDSII---------------------------------    515
SLC16A11        LLSPGGPG--STLDTTC-------------------------------------------    447
SLC16A12        --TNGSVAYSVARELDQKHGEPVATAV--------PGYSLT-------------------    516
SLC16A13        LEED--------------------------------------------------------    426
SLC16A14        ------HV----------------------------------------------------    510
AAEL000471      ------------------------------------------------------------    689


SLC16A1         ------    500
SLC16A2         ------    539
SLC16A3         ------    465
SLC16A4         ------    487
SLC16A5         GWNSPT    505
SLC16A6         ------    523
SLC16A7         ------    478
SLC16A8         ------    504
SLC16A9         ------    509
SLC16A10        ------    515
SLC16A11        ------    447
SLC16A12        ------    516
SLC16A13        ------    426
SLC16A14        ------    510
AAEL000471      ------    689
```

```
#
#
#  Percent Identity  Matrix - created by Clustal2.1
#
#
```

	SLC16A1	SLC16A2	SLC16A3	SLC16A4	SLC16A5	SLC16A6	SLC16A7	SLC16A8	SLC16A9	SLC16A10	SLC16A11	SLC16A12	SLC16A13	SLC16A14	AAEL000471
SLC16A1	100	23.73	44.57	23.99	29.07	28.48	59.21	39.43	24.54	23.74	28.15	28.72	30.24	25.12	26.15
SLC16A2	23.73	100	25.46	22.86	24.88	20.13	22.97	26.17	25.06	52.17	27.21	22.61	24.15	22.53	23.52
SLC16A3	44.57	25.46	100	25.37	36.81	30.7	46.74	56.93	28.75	25.81	34.27	34.68	33	27.54	26.62
SLC16A4	23.99	22.86	25.37	100	24.46	27.22	26.51	25.06	26.1	24.34	27.16	29.79	29.44	27.17	27.1
SLC16A5	29.07	24.88	36.81	24.46	100	28.89	32.16	37.5	24.76	22.88	30.68	29.57	29.24	24.34	24.88
SLC16A6	28.48	20.13	30.7	27.22	28.89	100	31.84	27.97	26.64	19.91	25.35	26.06	33.73	23.46	27.16
SLC16A7	59.21	22.97	46.74	26.51	32.16	31.84	100	42.77	27.1	24.55	32.41	30.22	31.5	27.23	26.88
SLC16A8	39.43	26.17	56.93	25.06	37.5	27.97	42.77	100	27.27	26.67	36.59	32.64	35.32	24.41	27.64
SLC16A9	24.54	25.06	28.75	26.1	24.76	26.64	27.1	27.27	100	26.89	26.97	27.1	28.89	32.16	27.53
SLC16A10	23.74	52.17	25.81	24.34	22.88	19.91	24.55	26.67	26.89	100	27.78	23.21	24.64	25.87	25.72
SLC16A11	28.15	27.21	34.27	27.16	30.68	25.35	32.41	36.59	26.97	27.78	100	31.28	46.93	26.76	28.02
SLC16A12	28.72	22.61	34.68	29.79	29.57	26.06	30.22	32.64	27.1	23.21	31.28	100	33.33	28.47	26.23

SLC16A13	30.24	24.15	33	29.44	29.24	33.73	31.5	35.32	28.89	24.64	46.93	33.33	100	27.14	25.81
SLC16A14	25.12	22.53	27.54	27.17	24.34	23.46	27.23	24.41	32.16	25.87	26.76	28.47	27.14	100	25.1
AAEL000471	26.15	23.52	26.62	27.1	24.88	27.16	26.88	27.64	27.53	25.72	28.02	26.23	25.81	25.1	100
