# Supplementary material for: Aedes aegypti dyspepsia encodes a novel member of the SLC16 family of transporters and is critical for reproductive fitness
Source: PLoS Negl Trop Dis. 2021 Apr 7;15(4):e0009334. doi: 10.1371/journal.pntd.0009334 (PMC8055033; doi:10.1371/journal.pntd.0009334)
Supplement: S4 Fig — (PDF) [file pntd.0009334.s004.pdf]

Transmembrane domains predicted by TOPCONS

Cytoplasmic loop (underlined)

AAEL000471	*Ae. aegypti*	ASIS005594	*An. sinensis*	AMEC005770	*An. melas*
AALF018415	*Ae. albopictus*	AARA017939	*An. arabiensis*	ADAC009811	*An. darlingi*
CPIJ002846	*Cx. quinquefasciatus*	AGAP003205	*An. gambiae*	CG8468	*D. melanogaster*

```
AAEL000471     ------MPPQNTIEMSTKPDAENGTKKAQN--GNT-NKLQMMKEEPPTTSVIVPPDGGWG    51
AALF018415     --------------MSTKPDAENGTKKVQN--GNTNNKLQLMKEEPPTTSVIVPPDGGWG    44
CPIJ002846     -------------MSTKQPDGENGTKKVQN--GDNK---QAKNAEPPTTSVVVPPDGGWG    42
ASIS005594     LIQQQRMPPQNTIELTTKPDGENGATGKLLAAN---GNGKVKEEDPPTTTLIVPPDGGWG    57
AARA017939     ------MPPQNSIELTTKPEAENGATGKLLANGNGNGDTEKAKEDPPTTTLIVPPDGGWG    54
AGAP003205     ------MPPQNSIELTTKPEAENGATGKLLANGNGNGDTEKAKEDPPTTTLIVPPDGGWG    54
AMEC005770     ------MPPQNSIELTIKPEAENGATGKLLANGNGNGDTEKAKEDPPTTTLIVPPDGGWG    54
ADAC009811     ------MPPQNSIELTTKPEA-DGANGKLLPTNGTNGKGSDGVEDPPTTTLIVPPDGGWG    53
CG8468_Dmel    ------MATQQ-----TKSQDELASKGLVSKQPENIKEENEGEEEIEEAAVVVPPDSGWA    49
                                :   .:.             .    :   ::::****.**.

AAEL000471     WLVMIASFLCNTVVDGIVFSAGMFQDPIRLDFGVGKAEVALVSSLLSGFYLLTGPFVSAL    111
AALF018415     WLVMIASFLCNTVVDGIVFSAGMFQDPIRLDFGVGKAEVALVSSLLSGFYLLTGPFVSAL    104
CPIJ002846     WLVMIASFLCNTVVDGIVFSAGMFQDPIRLDFGVGKAEVALVSSLLSGFYLLTGPFVSAL    102
ASIS005594     WLVMVASFCCNVVVDGIVMNVGSFLRPIRDEFGVSEAEVALVSSLLSGFYLLTGPFVSAL    117
AARA017939     WLVMVASFFCNVIVDGIVMNVGSFLGPIRDEFGVSEAEVALVSSLLSGFYLLTGPFVSAL    114
AGAP003205     WLVMVASFFCNVIVDGIVMNVGSFLGPIRDEFGVSEAEVALVSSLLSGFYLLTGPFVSAL    114
AMEC005770     WLVMVASFFCNVIVDGIVMNVGSFLGPIRDEFGVSEAEVALVSSLLSGFYLLTGPFVSAL    114
ADAC009811     WLVMVASFFCNVIVDGIVMNVGSILVPIGKEFNVSSTESALVGSLLSGFYLLTGPFVSAL    113
CG8468_Dmel    WVVMVASFLCCTVIDGIVFCSGLIQEQLMAEFGVSKAYVAFVSSLLSGCYLMAGPFVSAM    109
               *:**:*** * .::****:  * :   :  :*.*..:  *:*.***** **::******:

AAEL000471     ANRWGFRPVTIMGAVIASIGFGLSYYGTSLGYLYVTYGIIGGIGFCFIYVPSVITVGYYF    171
AALF018415     ANRWGFRPVTILGAVIASIGFGLSYYGTSLGYLYVTYGIIGGIGFCFIYVPSVITVGYYF    164
CPIJ002846     ANRWGFRPVTIIGAVIASIGFVLSYYAQSLGFLYVTYGVIGGIGFCFIYVPSVITVGYYF    162
ASIS005594     ANRWGFRIVTILGAVVAAFGFFVSQYATNIVFLYVTFGFIGGVGFCFIYVPSVITVGYYF    177
AARA017939     ANRWGFRIVTIIGSVVAAVGFFVSQYATSIVFLYVTFGFIGGVGFCFIYVPSVITVGYYF    174
AGAP003205     ANRWGFRIVTIIGSVVAAVGFFVSQYATSIVFLYVTFGFIGGVGFCFIYVPSVITVGYYF    174
AMEC005770     ANRWGFRIVTIIGAVVAAVGFFVSQYATSIVFLYVTFGFIGGVGFCFIYVPSVITVGYYF    174
ADAC009811     ANRWGFRIVTIIGAVVSAFGFFISMYATNIITLYITVGVIGGIGFCFIYVPSVITVGYYF    173
CG8468_Dmel    ANRFGFRPVTITGAIFAAICFGLSYFATSVEYLFLIYGVLGGIGFCMVYIPAVVIIGFYF    169
               ***:*** *** *::.::. * :* :. .:  *::  *.:**:***::*:*:*: :*:**

AAEL000471     EKWRALATGIALCGSGVGTFVFAPLSAMLIE-KFGWRGALLAQAAIILLCALFGCIFRPI    230
AALF018415     EKWRALATGIALCGSGVGTFVFAPLSAMLIE-KFGWRGALLAQAAIILLCALFGCIFRPI    223
CPIJ002846     EKWRALATGIALCGSGVGTFVFAPLSSMLIA-QFGWRGALLVQAAIILLCALFGCIFRPI    221
ASIS005594     EKWRAVATGIALCGSGVGTFIFSPVNAMLIE-SFGWRGALVAQAGIILSCIAYGAIFRPI    236
AARA017939     EKWRAVATGIALCGSGVGTFIFSPVNAMLIE-SLGWRSALVAQAAIILLCIACGAIFRPI    233
AGAP003205     EKWRAVATGIALCGSGVGTFIFSPVNAMLIE-SLGWRSALVAQAAIILLCIACGAIFRPI    233
AMEC005770     EKWRAVATGIALCGSGVGTFIFSPVNAMLIE-SLGWRSALVAQAAIILVCIACGAIFRPI    233
ADAC009811     EKWRAIATGVALCGSGVGTFIFAPLNKVLLGPNDNWRETLGYQAGIILSSILFALVFRPI    233
CG8468_Dmel    EKWRALATGVAMCGSGVGTFVFAPLTKILLK--SGWRYTLAIQGIIVLSCALFGLAFRPI    227
               *****:***:*:********:*:*:. :*:    .** :*  *. *:* .   .  ****

AAEL000471     QPIQVTITKDEDTPAEKGTLLGEGLP---------VVYTKPLPEGRFAYSVPNSSHSTWM    281
AALF018415     QPIQVTVTKDEDTPAEKGTLLGEGLP---------IVYTKPLPEGRFAYSVPNSSHSTWM    274
CPIJ002846     QPITVTLTKDEDTPAEKGSLLGDGLP---------VVYTRPLPEGRFAYSVPNSSHNTWM    272
ASIS005594     QPTEVTVTKDPDTPADKGIRLGEGLP---------TVYTRPLPEGRYAYSMPNSSHNTWM    287
AARA017939     QPTEVTATKAADTPADKGILLGEGLP---------VVYTRPLPEGRYAYSMPNSSHNTWM    284
AGAP003205     QPTEVTATKAADTPADKGILLGEGLP---------VVYTRPLPEGRYAYSMPNSSHNTWM    284
AMEC005770     QPTEVTATKAADTPADKGILLGEGLP---------VVYTRPLPEGRYAYSMPNSSHNTWM    284
ADAC009811     QPTEVTVKKDEDTPAEKGIRLGEGLP---------VVYTRPLPEGRYAYSMPNSSHNTWM    284
CG8468_Dmel    QPITLSVTNEEGDEQEKKKLNGHGNTVAPTPQLHQAAFTKPLPEGRFAYSMPNSAHNTYM    287
               **  :: .:  .   :*    *.*            .:*:******:***:***:*.*:*

AAEL000471     GVSPNTQYPTAAEVFR-GSGHNLERRPSNHSGLLTNENIQHTTKKLEQLSKIQKRLSGQM    340
AALF018415     GVSPNTQYPTAAEVFR-GSGHNLERRPSGHSGLLTNENIQHTTKKLEQLSKIQKRLSGQM    333
CPIJ002846     GASPNTQYPTAAEVFR-GSGHNLERRPSNQSGLLTHENIQTTTKKLEQLSKIQKRLAGQM    331
ASIS005594     GANPNYQYPTAAEIFRQGSGHNLDRRPSHTSGQLVSHNLQSTTKKLE---KIQKRLAGQM    344
AARA017939     GANPNYQYPTAAEIFRQGSGHNLDRRPSHTSGQLVSHNLQSTTKKLE---KIQKRLAGQM    341
AGAP003205     GANPNYQYPTAAEIFRQGSGHNLDRRPSHTSGQLVSHNLQSTTKKLE---KIQKRLAGQM    341
AMEC005770     GANPNYQYPTAAEIFRQGSGHNLDRRPSHTSGQLVSHNLQSTTKKLE---KIQKRLAGQM    341
ADAC009811     GANPNYQYPTAAEIFRQGSGHNLDRRPSHTSGQLVSHNLQSTTKKLE---KIQKRLAGQM    341
CG8468_Dmel    GASQRNHYPTAQEIFK---GMNLERRPSGTAQGSKGT-------ELKQLRKSQ-----PT    332
               *..  . :**** *:*:   * **:****  :             :*:   * *
```

```
AAEL000471      TPEDTIHAPRFPLPHHELVTVGEAAEEEETENGTLLTGEVKPQPVI--TTPSTRQRSHTV   398
AALF018415      TPEDTIHAPRFPLPHHELNTVGEAAEEEETENGTLLTGEVKPVPVV--STPSSRQRSHTV   391
CPIJ002846      TPEDTIHAPRFPIPHHELNTVGEAE-EEETENGTLLTGEQKTTLVVPTSAPGARQRSHTV   390
ASIS005594      TPDDTIHAHGFPTAHHELNPVGEAD-EEETENGTLLVAPEQQTITV---T-TASTRRHTV   399
AARA017939      TPDDTIHAHGYPTAHHELNPVGEAD-EEETENGTLLAAPEQQSVTI---T-TAAGRRHTV   396
AGAP003205      TPDDTIHAHGYPTAHHELNPVGEAD-EEETENGTLLAAPEQQSVTI---T-TAAGRRHTV   396
AMEC005770      TPDDTIHAHGYPTAHHELNPVGEAD-EEETENGTLLAAPEQQSVTI---T-TAAGRRHTV   396
ADAC009811      TPDDTIHAHGFQSAHHELNPVGEAD-EEDTENGTLLAAPEQTTITA---ATTTSARRHTV   397
CG8468_Dmel     TPNGDPQQLTF-NLHKELTTVGENEEE--AENDNLLETEAKP--------VTIQARRHTV   381
                **:.  :   :   *:**  ***   *  :**..**    :              * ***

AAEL000471      SGRRPNESGSRQGS-----RRGTLTDVTRPMYRDDIFFTGSLVRIPQYQSQTSLGYHMSV   453
AALF018415      SGRRPGEGGSRQGS-----RRGTLTDATRPLYRDDIFFTGSLVRIPQYQSQTSLGYHMSV   446
CPIJ002846      SGRRPNEAGSRQGS-----RRGTLTDGTRPMYRDDIFFTGSLVRIPQYQSTTSLGYHMSV   445
ASIS005594      SGRRPNEPASSRHG-----SRRTLNDGARPMYRDDIFFTGSLARIPQYQSQTSLGYHMSV   454
AARA017939      SGRRPNEPASSRHG-----SRRTLNDGARPMYRDDIFFTGSLARIPQYKSQTSLGYHMSV   451
AGAP003205      SGRRPNEPASSRHG-----SRRTLNDGARPMYRDDIFFTGSLARIPQYKSQTSLGYHMSV   451
AMEC005770      SGRRPNEPASSRHG-----SRRTLNDGARPMYRDDIFFTGSLARIPQYKSQTSLGYHMSV   451
ADAC009811      SGRRPNEPAGSRHG-----SRRTLNEGARPMYRDDIFFTGSLARIPQYQSQTSLGYHMSV   452
CG8468_Dmel     SGRRPQDIGKRSAAAAHEAHHGQSHSSSRPMYRDDIFFSGSLTRIPQYQSQTSLAYHMSV   441
                ***** : .    .      :    . :**:*******:***.*****:* ***.*****

AAEL000471      TRLPTQTDVEEIEEQSCKICPEAVRRTLATMLDMTLLKSPSFMLLAVSGFFTMMGFFVPF   513
AALF018415      TRLPTQTDVEEIEEQQCKICPEAVRRTLATMLDMTLLKSPSFMLLAISGFFTMMGFFVPF   506
CPIJ002846      TRLPTHTDVEEAEDQSCKICPEAVRRTLATMLDMTLLKSPSFMLLAVSGFFTMMGFFVPF   505
ASIS005594      TRLPTQADVEETETKQCMVCPEAVRRTLATMLDMSLLKSVSFMMLAFSGFLTMMGFFVPF   514
AARA017939      TRLPTQADVEETETEQCMVCPEAVRRTLVTMLDMSLLKSISFMMLAFSGFLTMMGFFVPF   511
AGAP003205      TRLPTQADVEETETEQCMVCPEAVRRTLVTMLDMSLLKSISFMMLAFSGFLTMMGFFVPF   511
AMEC005770      TRLPTQADVEETETEQCMVCPEAVRRTLVTMLDMSLLKSISFMMLAFSGFLTMMGFFVPF   511
ADAC009811      TRLPTQTDVEEEVEKQCKICPEAVRRTLATMLDMSLLKSPSFMMLAFSGFFTMMGFFVPF   512
CG8468_Dmel     TRLPTKQDMLEDRQKGCRICPEAVRRTLSTMLDTTLLKSPAFMCLAFSGFLTMMGFFVPF   501
                *****: *: *   : * :********* **** :**** :** **.***:*********

AAEL000471      MYITQRATTGGMDQNVALFIVSAIGISNTIARIVCGFLSSFKSVNALYINNVAITMGGIA   573
AALF018415      MYITQRATTGGMDQNVALFIVSAIGISNTIARIVCGFLSSFKSVNALWINNVAITMGGIA   566
CPIJ002846      MYISQRATSGGMDSNVALYIVSAIGISNTIARIVCGFLSSFKSVNALHLNNVAITMGGLA   565
ASIS005594      IYVKQRATAGGMAEDVSTFIVSAIGISNTIARVVCGLLTSFKDVNALHLNNVAITLGGIA   574
AARA017939      LYVKQRAVAGGMADEVSTFIVSAIGISNTIARVVCGVLTSFKNINALHLNNVAITLGGIA   571
AGAP003205      LYVKQRAVAGGMADEVSTFIVSAIGISNTIARVVCGVLTSFKNINALHLNNVAITLGGIA   571
AMEC005770      LYVKQRAVAGGMADEVSTFIVSAIGISNTIARVVCGVLTSFKNINALHLNNVAITLGGIA   571
ADAC009811      TYVKLRATGAGMADDVATFIVSAIGISNTIARIVCGLLSSFQSVNALHLNNVAITLGGIA   572
CG8468_Dmel     TFLVDRAKAAGMDEQAAYGVLSGIGVVNTFARIVCGSLSSFPAVKPLWLNNFALTAGGIA   561
                 ::  **  .**  .:.:  ::*.**: **:**:*** *:**  :: * :**.*:* **:*

AAEL000471      TMLSGLYITEAFQFTYAGIFGIAIACFSALRSILVVDLMGLEKLTNAFGILCLFQGMAAA   633
AALF018415      TMLSGLYITEAFQFTYAGIFGIAIACFSALRSILVVDLMGLEKLTNAFGILCLFQGMAAA   626
CPIJ002846      TMASGLYITEAYQFTYAAIFGLAIACFSALRSILVVDLMGLEKLTNAFGILCLFQGLAAA   625
ASIS005594      TMISGIYITEAFQFTYAGIFGIAIACFSALRSILVVDLMGLEKLTNAFGILCLFQGIAAF   634
AARA017939      TMMSGMYITEVYQFTYAAIFGLAIACFSALRSILVVDLMGLEKLTNAFGILCLFQGIAAF   631
AGAP003205      TMMSGMYITEVYQFTYAAIFGLAIACFSALRSILVVDLMGLEKLTNAFGILCLFQGIAAF   631
AMEC005770      TMMSGMYITEAYQFTYAAIFGLAIACFSALRSILVVDLMGLEKLTNAFGILCLFQGIAAF   631
ADAC009811      TMISGIYITEATQFTYAGIFGIAIACFSALRSILVVDLMGLEKLTNAFGILCLFQGVAAF   632
CG8468_Dmel     TICSGIVINTATQWTFALFFGICIACFSALRSLIAVELIGLEKLTNAYGFLMLFQGLAAT   621
                *: **: *. . *:*:* :**:.*********::.*:*:********:*:* ****:**

AAEL000471      IGAPIAGFFTDLTGSYNVSFYISGALITISAVLCYPLNMVSKWEKKRALNKKPGTV-      689
AALF018415      IGAPIAGFFTDLTGSYNVSFYISGALITISAVLCYPLNMVNRWERKRALKK-PGPV-      681
CPIJ002846      IGAPIAGFFTDLTGSYDVSFYISGALITISAVLCYPLNMVNKWEKKRATNKKVVPA-      681
ASIS005594      LGAPIAGYFTQLTGSYNASFYISGALITLSAVLCYPLGMVNRWEKQRAAKKDSQKI-      690
AARA017939      LGAPIAGYFTEMTGTYDASFYISGALITLSAVLCYPLAFVNRWEKQRAAKKDSQKV-      687
AGAP003205      LGAPIAGYFTELTGTYDASFYISGALITLSAVLCYPLAFVNRWEKQRAAKKDSQKV-      687
AMEC005770      LGAPIAGYFTELTGTYDASFYISGALITLSAVLCYPLAFVNRWEKQRAAKKDSQKV-      687
ADAC009811      VGTPLAGFFFGLTGTFDVSFYVSGGLITLSAILCYPLGYVNRWEKQRAAKKDSQKV-      688
CG8468_Dmel     FGSPIAGGLYVMTGSYNAAFYFAGGLILLSAFLCYPLTWVSRWEERRNEKLTPLPAA      678
                .*:*:** :  :**:::.:**.:*.** :**.*****  *.:**.:*  :
```

#
#
Percent Identity Matrix - created by Clustal2.1
#
#

	AAEL000471	AALF018415	CPIJ002846	ASIS005594	AARA017939	AGAP003205	AMEC005770	ADAC009811	CG8468
AAEL000471	100.00	96.62	89.38	74.89	73.35	73.50	73.50	71.45	57.73
AALF018415	96.62	100.00	88.92	75.45	73.33	73.48	73.48	71.56	58.47
CPIJ002846	89.38	88.92	100.00	74.66	73.15	73.29	73.44	71.22	57.67
ASIS005594	74.89	75.45	74.66	100.00	91.37	91.52	91.67	84.77	54.50
AARA017939	73.35	73.33	73.15	91.37	100.00	99.85	99.27	82.22	52.43
AGAP003205	73.50	73.48	73.29	91.52	99.85	100.00	99.42	82.36	52.28
AMEC005770	73.50	73.48	73.44	91.67	99.27	99.42	100.00	82.51	52.43
ADAC009811	71.45	71.56	71.22	84.77	82.22	82.36	82.51	100.00	55.02
CG8468	57.73	58.47	57.67	54.50	52.43	52.28	52.43	55.02	100.00
